# Encapsulation of redox polysulphides via chemical interaction with nitrogen atoms in the organic linkers of metal-organic framework nanocrystals

**DOI:** 10.1038/srep25555

**Published:** 2016-05-05

**Authors:** Jung Hyo Park, Kyung Min Choi, Dong Ki Lee, Byeong Cheul Moon, Sang Rim Shin, Min-Kyu Song, Jeung Ku Kang

**Affiliations:** 1 Department of Materials Science and Engineering, Advanced Institute of Science and Technology Korea 291 Daehak-ro, Yuseong-gu, Daejeon 34141, Republic of Korea; 2 Department of Chemical and Biological Engineering, Sookmyung Women’s University Cheonpa-ro 47-gil 100, Yongsan-gu, Seoul 04310, Republic of Korea; 3 Graduate School of EEWS (Energy, Environment, Water, and Sustainability) Korea Advanced Institute of Science and Technology 291 Daehak-ro, Yuseong-gu, Daejeon 34141, Republic of Korea; 4 School of Mechanical and Materials Engineering, Washington State University Pullman, Washington 99164-2920, USA

## Abstract

Lithium polysulphides generated during discharge in the cathode of a lithium-sulphur redox cell are important, but their dissolution into the electrolyte from the cathode during each redox cycle leads to a shortened cycle life. Herein, we use *in situ* spectroelectrochemical measurements to demonstrate that sp^2^ nitrogen atoms in the organic linkers of nanocrystalline metal-organic framework-867 (nMOF-867) are able to encapsulate lithium polysulphides inside the microcages of nMOF-867, thus helping to prevent their dissolution into the electrolyte during discharge/charge cycles. This encapsulation mechanism of lithiated/delithiated polysulphides was further confirmed by observations of shifted FTIR spectra for the C = N and C-N bonds, the XPS spectra for the Li-N bonds from nMOF-867, and a visualization method, demonstrating that nMOF-867 prevents lithium polysulphides from being dissolved in the electrolyte. Indeed, a cathode fabricated using nMOF-867 exhibited excellent capacity retention over a long cycle life of 500 discharge/charge cycles, with a capacity loss of approximately 0.027% per cycle from a discharge capacity of 788 mAh/g at a high current rate of 835 mA/g.

The most critical challenge in energy storage is the development of high-performance energy storage devices that combine a stable cycle life with a high energy density^1^,^2^,^3^,^4^,^5^,^6^. The lithium-sulphur (Li-S) redox cell, which has a high theoretical energy density, may be a good system that can overcome this challenge^7^,^8^. In addition, sulphur is safe, inexpensive and abundant on Earth^9^,^10^. Unfortunately, the high-order polysulphides formed by reactions between cyclic octasulphur (S_8_) and lithium ions during the discharge reactions are soluble in most organic electrolyte solutions^11^,^12^, thus leading to quickly fading capacities during discharge/charge redox cycles. Moreover, the lithium polysulphides can move to the lithium anode electrode and form insoluble Li_2_S_2_ and/or Li_2_S species on its surface^13^,^14^. Furthermore, the lithium polysulphides can diffuse back and forth between the cathode and anode electrodes, in a phenomenon known as the shuttle effect, thus decreasing the cycle life of Li-S redox cells^15^,^16^. Therefore, a new paradigm structure or mechanism that can effectively prevent the dissolution of polysulphides to provide excellent capacity retention over long discharge/charge redox cycles would represent a major breakthrough in the realization of high-performance energy storage.

In principle, the functional heteroatoms embedded in the organic linkers in metal-organic frameworks (MOFs)^17^,^18^,^19^ can be used to create specific interactions with the lithium polysulphides^20^,^21^. Therefore, we hypothesized that the sp^2^ nitrogen in the linkers of the microcages of nanosized MOF-867^22^ (Zr_6_O_4_(OH)_4_(BPYDC)_6_, BPYDC = 2,2′-bipyridine-5,5′-dicarboxylate, abbreviated as nMOF-867) could be combined with the lithium polysulphides to prevent their dissolution into the electrolyte, as shown in Fig. 1. This combination would allow full redox reactions with sulphur over an extended cycle life. For comparison, we also prepared another nanosized MOF with no sp^2^ nitrogens in its organic linker (Zr_6_O_4_(OH)_4_(BPDC)_6_, BPDC = 4,4′-biphenyldicarboxylate, termed nUiO-67)^23^ but with the same crystal structure as nMOF-867. We found that the high-order polysulphides from the lithiation of the S_8_ species could be encapsulated inside the microcages of nMOF-867, thus preventing lithium polysulphide dissolution and extending the cycle life. To confirm the self-encapsulation of polysulphides in the microcages, we used a combination of experimental characterizations including *in situ* spectroelectrochemical measurements, FTIR spectroscopy to identify C = N and C-N bonds, XPS spectroscopy to determine the existence of Li-N bond orbitals during discharge, and a visualization method. This is the first direct demonstration confirming the mechanism and interaction of lithium polysulphide with functional heteroatoms in the organic linkers of MOFs.

## Results and Discussion

nMOF-867 was prepared by dissolving zirconium chloride (ZrCl_4_) and H_2_BPYDC in *N,N*-dimethylformamide (DMF) in a 20 mL glass vial at room temperature. The sp^2^ nitrogen in BPYDC exists as a heteroatom replacing the = CH- group in the aromatic ring. Then, acetic acid and triethylamine (TEA) were added to the stock solution, which was sonicated for 20 min. The glass vial was placed into an oven at 85 °C for 12 hrs to grow the nanocrystalline MOFs. For nUiO-67, a H_2_BPDC organic linker was used in place of H_2_BPYDC in nMOF-867, and all of the other ingredients as well as the heating process were the same as those used for nMOF-867 (Figure S1). The products were washed with DMF several times and immersed in methanol for three days. Next, the products were activated in a vacuum oven at 100 °C for 24 hrs. Then, the activated nMOFs were mixed with high-purity sulphur in a mortar in an Ar-filled glove box, and the mixtures were placed in a sealed vessel. The vessel was heated to 155 °C for 12 hrs in a tubular quartz furnace with flowing Ar. The viscosity of sulphur was lowest at 155 °C, and the molten sulphur was found to be infused into the microcages of nMOFs. The sulphur-infiltrated nMOF-867 and nUiO-67 (abbreviated as nMOF-867/S and nUiO-67/S, respectively) were maintained in an Ar-filled glove box to avoid moisture uptake from the atmosphere.

**Figure 2:**
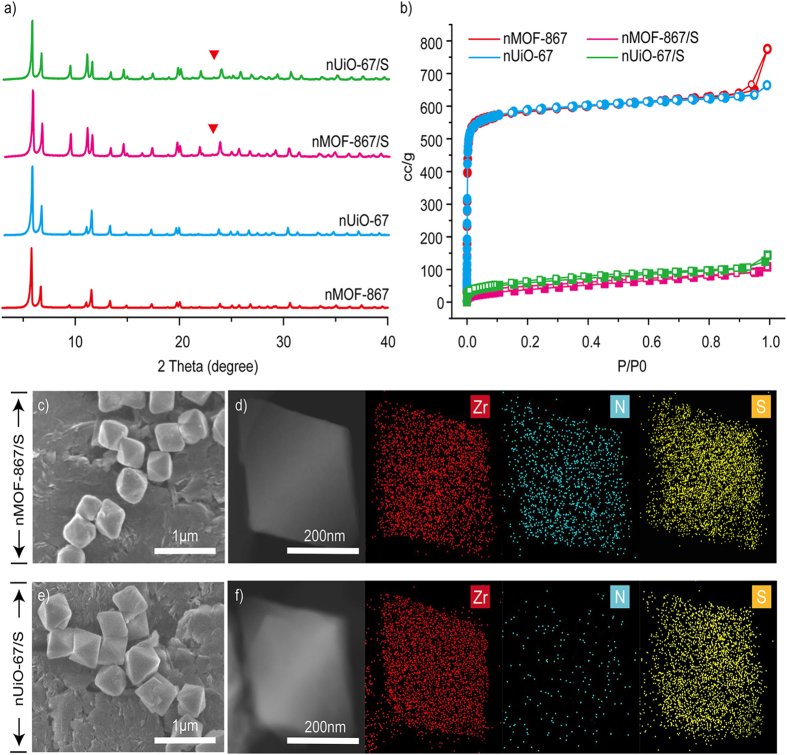
Structural analysis of pristine nMOF and nMOF/sulphur composites using PXRD, nitrogen gas adsorption, and STEM and EDS mapping. (**a**) The PXRD patterns of pristine nMOF-867, pristine nUiO-67, nMOF-867/S and nUiO-67/S. (**b**) Nitrogen gas adsorption of pristine nMOF-867, pristine nUiO-67, nMOF-867/S and nUiO-67/S. (**c**,**e**) SEM images of nMOF-867/S and nUiO-67/S. (**d**,**f**) EDS mapping of nMOF-867 and nUiO-67/S, respectively.

The structures were characterized by powder X-ray diffraction (PXRD), nitrogen gas adsorption isotherm measurements, scanning electron microscopy (SEM), scanning transmission electron microscopy (STEM), energy dispersive X-ray spectroscopy (EDS) and thermal gravimetric analysis (TGA). The PXRD patterns for nMOF-867 and nUiO-67 contained sharp diffraction peaks at defined positions that were in agreement with simulated patterns^24^,^25^, indicating that the samples possessed high crystallinity, and their underlying crystal structures were the same (Fig. 2a). The type I nitrogen gas adsorption isotherm (Fig. 2b) indicated that the permanent porosity and high BET surface areas (2250 m^2^/g for nMOF-867 and 2256 m^2^/g for nUiO-67) provided sufficient space for retaining the sulphur. The octahedral morphologies of nMOF-867 and nUiO-67 that were *ca*. 500 nm in diameter were also determined through SEM measurements (Figure S2). Notably, the fabrication of nMOF-867 and nUiO-67 particles that are homogeneous in size and morphology can eliminate the issues related to the transport properties of sulphur, lithium ions, and electrons^26^.

Before the infiltration of sulphur into the nMOFs, nMOF-867 and nUiO-67 were mixed with sulphur without a heating process. The measured PXRD patterns, which are shown in Figures S3 and S4, contained the main peak for pure sulphur at approximately 23° as well as the sharp peaks for nMOFs. In addition, the PXRD patterns after the heating process at 155 °C (Fig. 2a) demonstrated that nMOF-867/S and nUiO-67/S retained their crystal structures. However, the main peak for pure sulphur (indicated as the red triangle in the PXRD patterns shown in Fig. 2a) disappeared after the heating process because the sulphur particles were well absorbed into the microcages of the nMOF-867 and nUiO-67 particles^27^. In addition, after sulphur infiltration, the specific surface areas of nMOF-867/S and nUiO-67/S substantially decreased to 147 and 150 m^2^/g, respectively (Fig. 2b). In combination with the PXRD patterns, this evidence indicates that these surface area reductions did not result from structural collapse but were due to the infiltration of sulphur into the microcages of nMOF-867 and nUiO-67. The SEM images shown in Fig. 2c,e further indicated that both the crystal size and shape of nMOF-867/S and nUiO-67/S were well maintained after sulphur infiltration. Moreover, the STEM and EDS mapping (Fig. 2d,f) of nMOF-867/S and nUiO-67/S indicated that sulphur had infiltrated into both MOFs after the heating process. The nitrogen signal was detected only in nMOF-867/S because its organic linkers (BPYDC) contain sp^2^ nitrogen atoms. Moreover, the TGA measurements (Figures S5 and S6) of nMOF-867 and nUiO-67 demonstrated their identically high thermal stability up to 400 °C.

We also explored the electrochemical performance of the Li-S redox system with nMOFs/S. First, the nMOFs/S was mixed with conductive super P and polyvinylidene fluoride (PVDF) binder in *N*-methyl-2-pyrrolidinone (NMP), which resulted in the formation of a viscous slurry that was coated on aluminium foil and dried under vacuum at 60 °C for 24 hrs. Next, 2032-type cells were assembled with lithium foil as the counter electrode in an Ar-filled glove box. The electrolyte consisted of 1 M lithium bis(trifluoromethanesulphonyl)imide (LiTFSI) in *N*-methyl-*N*-butylpyrrolidinium bis(trifluoromethane sulphonyl)imide (PYR14TFSI)/1,2-dimethoxyethane/1,3-dioxolane (2:1:1 by volume) with LiNO_3_ (1 wt%) as an additive to help passivate the surface of the lithium anode and prevent the shuttle effect^28^,^29^.

**Figure 3:**
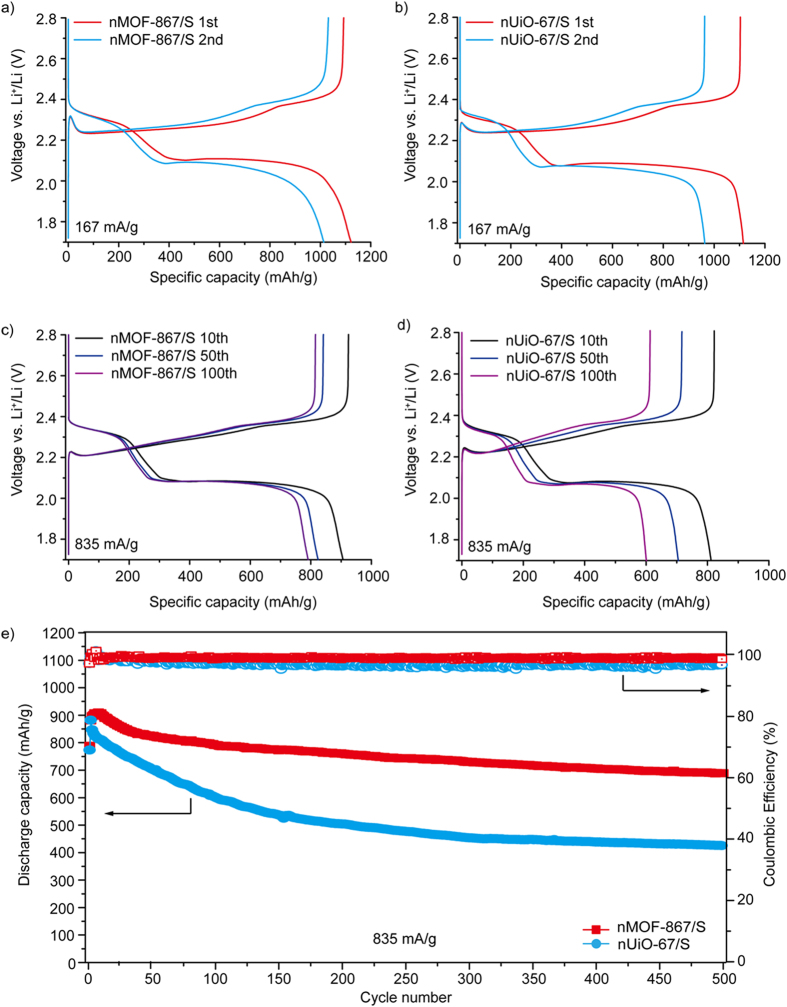
Electrochemical performance of nMOF-867/S and nUiO-67/S. (**a**) 1^st^ and 2^nd^ discharge/charge profiles of nMOF-867/S (at 167 mA/g). (**b**) 1^st^ and 2^nd^ discharge/charge profiles of nUiO-67/S (at 167 mA/g). (**c**) 10^th^, 50^th^ and 100^th^ discharge/charge profiles of nMOF-867/S (at 835 mA/g). (**d**) 10^th^, 50^th^ and 100^th^ discharge/charge profiles of nUiO-67/S (at 835 mA/g). (**e**) Long-term cycle performance over 500 cycles for nMOF-867/S and nUiO-67/S with a constant current of 835 mA/g.

The typical discharge/charge profiles of the nMOF-867/S composites are shown in Fig. 3a. Initially, a constant current of 167 mA/g, corresponding to 0.1 C, was used to study discharge/charge behaviours for nMOF-867 over a voltage range of 1.7 to 2.8 V. Two plateaus were observed at approximately 2.3 and 2.1 V in the discharge process. The plateau at approximately 2.3 V was related to the reduction of S_8_ to long-chain lithium polysulphides (Li_2_S_n_, 4 ≤ n ≤ 8), and the plateau at approximately 2.1 V corresponded to the formation of short-chain lithium polysulphides, such as Li_2_S_2_ and Li_2_S^30^,^31^. The initial discharge capacity of nMOF-867/S was 1121 mAh/g. For nUiO-67/S, the initial discharge behaviour and capacity (1115 mAh/g at 167 mA/g) were very similar to those of nMOF-867/S (Fig. 3b). Figure S7 also shows the first few discharge/charge profiles of nMOFs/S, in which the profiles for the 4^th^ to 8^th^ cycles were similar to those of the 1^st^ and 2^nd^ cycles. This result indicated that the N atoms covalently bound to C atoms in the organic linkers of the nanocrystalline MOFs formed secondary interactions with lithium polysulphides. In addition, nMOF-867/S (Fig. 3c) exhibited capacities of 906, 824 and 790 mAh/g for the 10^th^, 50^th^ and 100^th^ discharge capacities, respectively, at a high current rate of 835 mA/g. However, nUiO-67/S (Fig. 3d) exhibited capacities of 811, 704 and 600 mAh/g, respectively. Even after a long cycle life with more than 500 discharge/charge cycles, nMOF-867/S exhibited stable capacity retention with an average capacity loss of approximately 0.027% per cycle. Furthermore, our calculation for the number of N involved in nMOF-867 indicated that each nitrogen would form secondary interactions with multiple Li_2_S species when S_8_ was completely converted to 8 Li_2_S.

The nitrogen forms a strong covalent bond to carbon in the organic linkers (BPYDC, chemical formula = C_12_H_6_N_2_O_4_) of the MOF. Indeed, the results in Fig. 3c,e indicated that the electrochemical behaviour of MOF-867 was very stable over long cycles, thus implying that the BPYDC linkers in nMOF-867 were not destroyed but maintained the sp^2^ nitrogen in their aromatic rings over repeated discharge/charge cycles. Notably, excellent performance with a specific energy density of ~1700 Wh/g over 500 cycles at a high current rate of 835 mA/g was observed. Moreover, the capacity retention behaviours of nMOF-867 were maintained at high areal loadings of active materials up to 5 mg/cm^2^ (Figure S8). nMOF-867 with sp^2^ nitrogen atoms in its organic linkers provided stable capacity retention along with an excellent coulombic efficiency over a long discharge/charge cycle life for Li-S batteries. Therefore, the simple introduction of heterogeneous atoms into the organic linkers in the microcages of MOFs leads to stable energy performance with a high capacity retention over a long cycle life.

Additionally, we tested another set of Zn-based IRMOF-10s (ZnO_4_(BPDC)_3_ and ZnO_4_(BPYDC)_3_), which were separately prepared with BPDC, which has no sp^2^ nitrogens, and BPYDC, which has sp^2^ nitrogens in its linker^32^,^33^. The measured electrochemical performance (Figure S9) indicated that the capacity retention for the IRMOF-10 with the sp^2^ nitrogens (i.e., 64%) was much higher than for the IRMOF-10 with no sp^2^ nitrogens (i.e., 27%). These results confirmed that the sp^2^ nitrogen in the linker of the MOF structure enhanced the capacity retention over repeated discharge/charge cycles. Therefore, these results demonstrate that the simple introduction of heterogeneous atoms into the organic linkers in the microcages of MOFs can lead to excellent energy storage performance, thus opening a new route for providing an excellent solution for current energy storage issues.

We further investigated the discharge/charge behaviour by using Fourier transform infrared (FTIR) spectroscopy and X-ray photoelectron spectroscopy (XPS), and these results provided detailed information regarding the chemical binding of sp^2^ nitrogen atoms with polysulphides. Moreover, a visual verification method was used to directly observe the interactions between sp^2^ nitrogen atoms and polysulphides in the electrolyte. In addition, spectroelectrochemical measurements were performed with *in situ* observation of the chemical interactions during the discharge/charge processes. To identify the interaction, we prepared a polysulphide species (Li_2_S_4_), which was synthesized by mixing stoichiometric amounts of Li_2_S and sulphur in tetraethylene glycol dimethyl ether (TEGDME)^34^. Then, nMOF-867 and nUiO-67 were directly mixed with the polysulphide (Li_2_S_4_) solution. Finally, the products were washed with methanol to remove excess polysulphide molecules, and this was followed by drying in a vacuum oven at 60 °C for 24 hrs.

**Figure 4:**
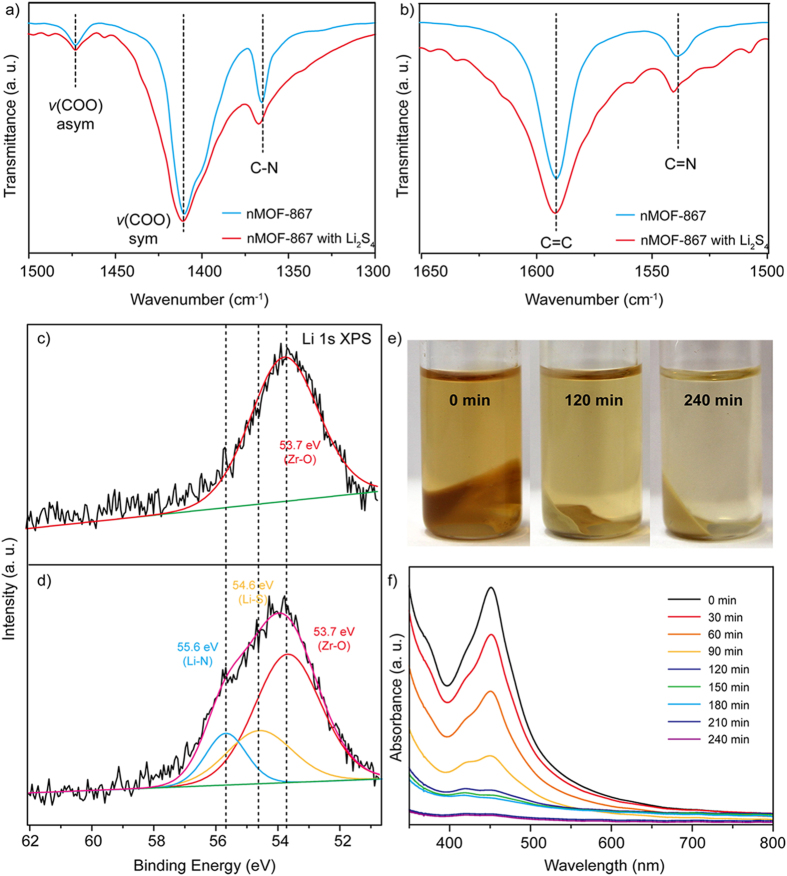
Investigation of the chemical interaction between the nitrogen atoms and the polysulphides. **(a**,**b**) FTIR spectra of pristine nMOF-867 and nMOF-867 with Li_2_S_4_. (**c**) XPS spectrum of pristine nMOF-867. (**d**) XPS spectrum of nMOF-867/Li_2_S_4_. (**e**) Photos show the colour changes of nMOF-867/Li_2_S_4_ over 240 min. (**f**) Absorbance intensity of UV-visible spectra for nMOF-867/Li_2_S_4_ every 30 min.

The FTIR spectra of nMOF-867 and nMOF-867/Li_2_S_4_ are shown in Figure S10, and partial ranges of the spectra are shown in Fig. 4a,b. The double bond stretching of C = C (1591 cm^−1^), the asymmetric and symmetric stretching modes of the carboxylate (-COO-, 1473 and 1410 cm^−1^), and the single bond stretching of C-O (1245 cm^−1^) and C-C (1163 cm^−1^) (Figure S11) indicated that the spectra of both samples did not change after interaction with Li_2_S_4_^35^. However, Fig. 4a,b show that the peaks related to atomic nitrogen (N *sp*^2^ orbitals) shifted from 1537 to 1541 cm^−1^ for the C = N double bonds and 1364 to 1367 cm^−1^ for the C-N single bond because the polysulphides undergo chemical interactions with the sp^2^ nitrogen atoms, which shift its stretching frequency. Moreover, to obtain more detail on the chemical binding of the nitrogen sp^2^ orbitals during lithiation, we also determined the XPS spectra (Fig. 4c,d) of pristine nMOF-867 and nMOF-867/Li_2_S_4_. The XPS spectrum of nMOF-867 in the absence of Li_2_S_4_ (Fig. 4c) was fitted to a single peak that corresponded to a Zr-O binding energy of 53.7 eV^36^. After chemical binding of nMOF-867 with Li_2_S_4_, the XPS spectra were deconvoluted into three peaks corresponding to Zr-O (53.7 eV), Li-N (55.6 eV) and Li-S (54.6 eV). Figure 4d^37^, which indicate that the lone pair of electrons in the sp^2^ nitrogen orbitals interacted with the Li in Li_2_S_4_.

Additionally, we developed a visualization method by mixing nMOF-867 and nUiO-67 with a 0.1 M Li_2_S_4_ TEGDME solution for up to 240 min. Because Li_2_S_4_ in solution is yellow, we were able to visualize the migration of Li_2_S_4_ by following the colour change. The photos show the colour changes of the Li_2_S_4_ solution after mixing with both MOFs at regular intervals of 0 min, 120 min and 240 min. The results in Fig. 4e indicate that the Li_2_S_4_ solution became transparent as Li_2_S_4_ was captured by MOF-867, and the nUiO-67 in the Li_2_S_4_ solution maintained its yellow colour even after 240 min (Figure S12a). These results were quantified through UV-Vis spectroscopic analysis of the Li_2_S_4_ solution after sedimentation of nMOFs every 30 min. The results demonstrated that the Li_2_S_4_ solutions that were mixed with both nMOFs initially exhibited high absorbance intensities in the wavelength range of 400 to 500 nm. As time progressed, the absorbance of the Li_2_S_4_ solutions mixed with nMOF-867 decreased substantially as the Li_2_S_4_ was encapsulated into nMOF-867 (Fig. 4f). However, the effect observed for nUiO-67 was much smaller (Figure S12b). These results imply that the sp^2^ nitrogen atoms in the organic linkers of nMOF-867 can encapsulate Li_2_S_4_ via chemical interactions, which prevent the dissolution of polysulphide into the electrolyte. Moreover, we performed additional experiments (Figure S13) to provide additional insight into the colour change. Initially, only nMOF-867 was placed in the TEGDME solvent in the absence of lithium polysulphides and stirred for 240 min. No colour change was observed after stirring for 240 min. Next, another glass vial was prepared containing only 0.1 M Li_2_S_4_ (lithium polysulphides) in the TEGDME solution. The initial colour of Li_2_S_4_ was yellow, and this colour was maintained after stirring for 240 min. These results provide clear evidence that the colour change was due to chemical interactions between sp^2^ nitrogens and lithium polysulphides and not to photooxidation.

**Figure 5:**
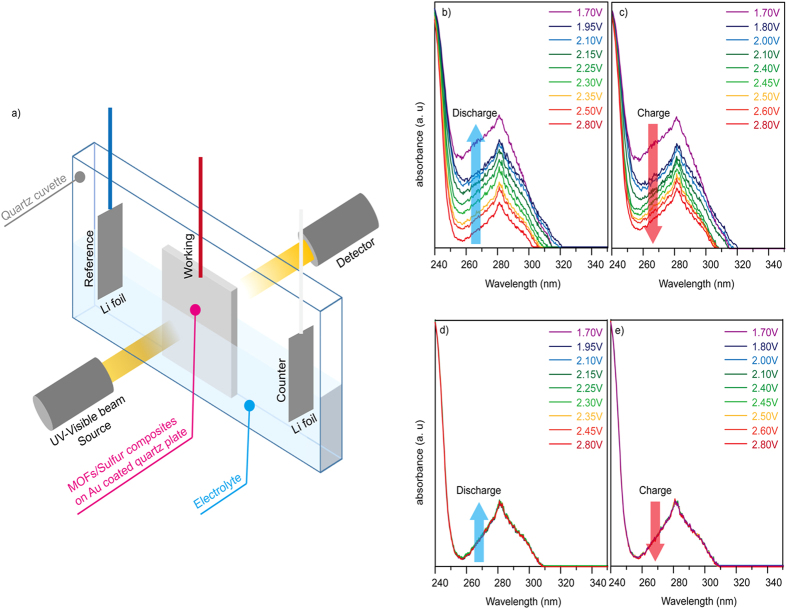
*In situ* spectroelectrochemical measurements. (**a**) Schematic illustration of instrument used for *in situ* UV-visible spectroscopy. (**b**,**c**) Change in the absorbance intensity of nMOF-867/S during the discharge/charge reactions. (**d**,**e**) Change in the absorbance intensity of nUiO-67/S during the discharge/charge reactions.

In addition, an *in situ* spectroelectrochemical method using a UV-visible instrument (DH-2000, Ocean Optics) linked to a potentiostat (SP-300, Bio-Logic) was developed. The cell system (Fig. 5a) was equipped with Li metal as the counter/reference electrode, and nMOFs/sulphur was deposited on Au-coated quartz plates as the working electrode. The Au-coated quartz plates were irradiated with UV and visible light to record the cyclic voltammetry (CV) data. We used quartz plates that barely absorb UV light and coated them with a thin film of Au to improve the conductivity. The UV-visible spectra of nMOF-867/S and nUiO-67/S were obtained at different voltage points on their CV profiles (Figures S14 and S15). Absorption peaks in the UV-Vis region (Fig. 5) were observed in both nMOF-867/S and nUiO-67/S during the discharge and charge cycles. The absorption intensities of nMOF-867/S (Fig. 5b) increased during the discharge reactions and returned to their original intensities during the charge reactions (Fig. 5c). The absorption intensities of nUiO-67/S remained unchanged (Fig. 5d,e). From these results along with those from the FTIR, XPS, and visualization verification analyses, we conclude that polysulphides are generated and encapsulated in the microcages of nMOF-867, which exhibited an *in situ* change in the UV-Vis absorption intensity due to light scattering from the polysulphides during the discharge/charge cycles^38^,^39^. In addition, nUiO-67 exhibited no change in its UV-Vis absorption because the generated polysulphides were easily dissolved in the electrolyte.

## Methods

### Synthesis of nMOF-867 and nUiO-67

For nMOF-867, zirconium chloride and acetic acid were dissolved in DMF. Simultaneously, BPYDC and triethylamine were dissolved in DMF. The solutions containing metal ions and organic linkers were combined in glass vials, which were tightly sealed and placed into an oven for 12 hrs. The white product was washed three times with DMF using a centrifuge and sonication. After the washing process, nMOF-867 was immersed in methanol for three days, with refreshing of the methanol. Finally, nMOF-867 was activated by removing the solvent in a vacuum oven. For nUiO-67, all of the procedures were the same as those used for nMOF-867 except that the organic linkers were exchanged with BPCD, and the reaction time was 6 hrs.

### Cathode preparation using nMOF/sulphur composites

The dried nMOF-867 or nUiO-67 was mixed with sulphur in a mortar. The mixtures were deposited into a sealed vessel that was placed into a quartz tubular furnace and heated to 155 °C for 12 hrs under an Ar flow. For the viscous slurry, nMOF-867/S (or nUiO-67/S) was mixed with carbon black (Super P) and polyvinylidene fluoride binder in *N*-methyl-2-pyrrolidinie. The well mixed slurry was cast on aluminium foil using a doctor blade and dried in a vacuum oven at 60 °C for 12 hrs. The entire sample preparation including mixing of the powders were conducted in an Ar-filled glove box with a low humidity below 1 ppm.

### Electrochemical measurements

1 M lithium bis(trifluoromethanesulphonyl)imide (LiTFSI) in *N*-methyl-*N*-butylpyrrolidinium bis(trifluoromethane sulphonyl)imide (PYR14TFSI)/1,2-dimethoxyethane/1,3-dioxolane (2:1:1 by volume) with LiNO_3_ (1 wt%) was used as the electrolyte. The electrochemical measurements were carried out using 2032-tyte coin cells, and lithium foil was used as the counter/reference electrode. Polypropylene membranes (Celgard Inc.) were used as separators. Galvanostatic discharge/charge tests were performed by cycling between 1.7 and 2.8 V at 167 mA/g and 835 mA/g using an electrochemical redox cycler (WBS3000, Wonatech). Moreover, the capacity retention behaviours of nMOF-867 were investigated by varying the loading amount of the active materials from 3.6 to 5 mg/cm^2^ (Figure S8).

## Additional Information

**How to cite this article**: Park, J. H. *et al*. Encapsulation of redox polysulphides via chemical interaction with nitrogen atoms in the organic linkers of metal-organic framework nanocrystals. *Sci. Rep*. **6**, 25555; doi: 10.1038/srep25555 (2016).

## Supplementary Material

Supplementary Information

## Figures and Tables

**Figure 1:**
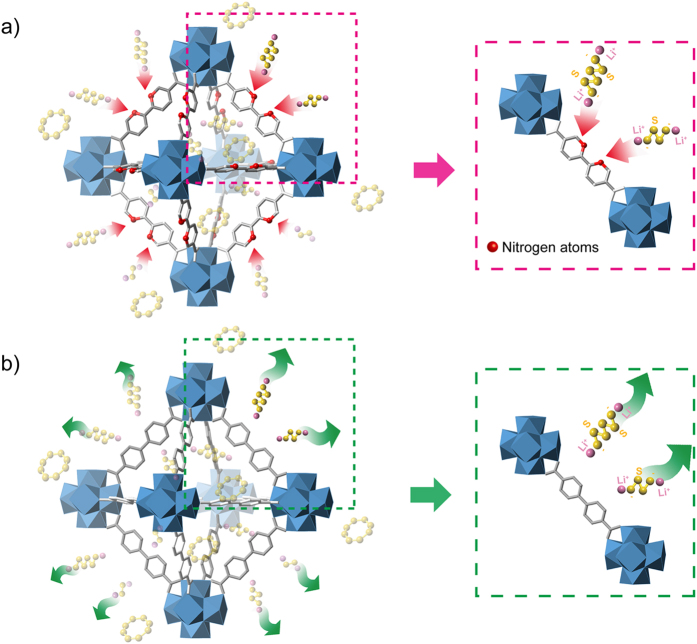
Schematic illustration of the chemical interactions between nitrogen atoms and lithium polysulphides. (**a**) The sp^2^ nitrogen atoms and lithium polysulphides interact chemically in nMOF-867. (**b**) The lithium polysulphides are dissolved into the electrolyte in nUiO-67.

## References

[b1] Manthiram A. . Rechargeable Lithium-Sulfur Batteries. Chem. Rev. 114, 11751–11787 (2014). 25026475 10.1021/cr500062v

[b2] Van Noorden R. Sulphur back in vogue for batteries. Nature 498, 416–417 (2013). 23803819 10.1038/498416a

[b3] Ji X. L., Lee K. T. & Nazar L. F. A highly ordered nanostructured carbon-sulphur cathode for lithium-sulphur batteries. Nat. Mater. 8, 500–506 (2009). 19448613 10.1038/nmat2460

[b4] Evers S. & Nazar L. F. New Approaches for High Energy Density Lithium-Sulfur Battery Cathodes. Accounts Chem. Res. 46, 1135–1143 (2013). 10.1021/ar3001348 23054430

[b5] Yin Y. X., Xin S., Guo Y. G. & Wan L. J. Lithium-Sulfur Batteries: Electrochemistry, Materials, and Prospects. Angew. Chem. Int. Ed. 52, 13186–13200 (2013). 10.1002/anie.201304762 24243546

[b6] Bresser D., Passerini S. & Scrosati B. Recent progress and remaining challenges in sulfur-based lithium secondary batteries - a review. Chem. Commun. 49, 10545–10562 (2013). 10.1039/c3cc46131a 24100379

[b7] Su Y. S., Fu Y. Z., Cochell T. & Manthiram A. A strategic approach to recharging lithium-sulphur batteries for long cycle life. Nat. Commun. 4, 8 (2013). 10.1038/ncomms3985 24346483

[b8] Ji X. L., Evers S., Black R. & Nazar L. F. Stabilizing lithium-sulphur cathodes using polysulphide reservoirs. Nat. Commun. 2, 7 (2011). 10.1038/ncomms1293 21610728

[b9] Manthiram A., Chung S. H. & Zu C. X. Lithium-Sulfur Batteries: Progress and Prospects. Adv. Mater. 27, 1980–2006 (2015). 25688969 10.1002/adma.201405115

[b10] Rosenman A. . Review on Li-Sulfur Battery Systems: an Integral Perspective. Adv. EnergyMater. 5, 21 (2015).

[b11] Barghamadi M. . Lithium-sulfur batteries-the solution is in the electrolyte, but is the electrolyte a solution? Energy Environ. Sci. 7, 3902–3920 (2014).

[b12] Lin Z. & Liang C. D. Lithium-sulfur batteries: from liquid to solid cells. J. Mater. Chem. A 3, 936–958 (2015).

[b13] Fang X. & Peng H. S. A Revolution in Electrodes: Recent Progress in Rechargeable Lithium-Sulfur Batteries. Small 11, 1488–1511 (2015). 25510342 10.1002/smll.201402354

[b14] Xu G. Y. . High performance lithium-sulfur batteries: advances and challenges. J. Mater. Chem. A 2, 12662–12676 (2014).

[b15] Zhang S. S. Liquid electrolyte lithium/sulfur battery: Fundamental chemistry, problems, and solutions. J. Power Sources 231, 153–162 (2013).

[b16] Xu R. . Role of Polysulfides in Self-Healing Lithium-Sulfur Batteries. Adv. Energy Mater. 3, 833–838 (2013).

[b17] Demir-Cakan R. . Cathode Composites for Li-S Batteries via the Use of Oxygenated Porous Architectures. J. Am. Chem. Soc. 133, 16154–16160 (2011). 21882857 10.1021/ja2062659

[b18] Wu H. B. . Embedding Sulfur in MOF-Derived Microporous Carbon Polyhedrons for Lithium-Sulfur Batteries. Chem.-Eur. J. 19, 10804–10808 (2013). 23801541 10.1002/chem.201301689

[b19] Xi K. . Carbon with hierarchical pores from carbonized metal-organic frameworks for lithium sulphur batteries. Chem. Commun. 49, 2192–2194 (2013). 10.1039/c3cc38009b 23396518

[b20] Seh Z. W. . Sulphur-TiO_2_ yolk-shell nanoarchitecture with internal void space for long-cycle lithium-sulphur batteries. Nat. Commun. 4, 6 (2013). 10.1038/ncomms2327 23299881

[b21] Kim J. W., Ocon J. D., Park D. W. & Lee J. Functionalized Graphene-Based Cathode for HighlyReversible Lithium-Sulfur Batteries. Chemsuschem 7, 1265–1273 (2014). 24464910 10.1002/cssc.201300782

[b22] Choi K. M. . Supercapacitors of Nanocrystalline Metal-Organic Frameworks. Acs Nano 8, 7451–7457 (2014). 24999543 10.1021/nn5027092

[b23] Chavan S. . H_2_ storage in isostructural UiO-67 and UiO-66 MOFs. Phys. Chem. Chem. Phys. 14, 1614–1626 (2012). 22187720 10.1039/c1cp23434j

[b24] DeCoste J. B. . Stability and degradation mechanisms of metal-organic frameworks containing the Zr6O4(OH)4 secondary building unit. J. Mater. Chem. A 1, 5642–5650 (2013).

[b25] Katz M. J. . A facile synthesis of UiO-66, UiO-67 and their derivatives. Chem. Commun. 49, 9449–9451 (2013). 10.1039/c3cc46105j 24008272

[b26] Zhou J. W. . The impact of the particle size of a metal-organic framework for sulfur storage in Li-S batteries. J. Mater. Chem. A 3, 8272–8275 (2015).

[b27] Zhou J. W. . Rational design of a metal-organic framework host for sulfur storage in fast,long-cycle Li-S batteries. Energy Environ. Sci. 7, 2715–2724 (2014).

[b28] Song M. K., Zhang Y. G. & Cairns E. J. A Long-Life, High-Rate Lithium/Sulfur Cell: A Multifaceted Approach to Enhancing Cell Performance. Nano Lett. 13, 5891–5899 (2013). 24219588 10.1021/nl402793z

[b29] Feng X. F. . Understanding the degradation mechanism of rechargeable lithium/sulfur cells: a comprehensive study of the sulfur-graphene oxide cathode after discharge-charge cycling. Phys. Chem. Chem. Phys. 16, 16931–16940 (2014). 24781200 10.1039/c4cp01341g

[b30] Zhou G. M. . A Graphene-Pure-Sulfur Sandwich Structure for Ultrafast, Long-Life Lithium-Sulfur Batteries. Adv. Mater. 26, 625–631 (2014). 24458578 10.1002/adma.201302877

[b31] Huang J. Q. . Ionic shield for polysulfides towards highly-stable lithium-sulfur batteries. Energy Environ. Sci. 7, 347–353 (2014).

[b32] Li H., Eddaoudi M., O’Keeffe M. & Yaghi O. M. Design and synthesis of an exceptionally stable and highly porous metal-organic framework. Nature 402, 276–279 (1999).

[b33] Huh S. . Two-dimensional metal-organic frameworks with blue luminescence. Dalton Trans. 39, 1261–1265 (2010). 20104352 10.1039/b916176g

[b34] Yu X. W. & Manthiram A. A class of polysulfide catholytes for lithium-sulfur batteries: energy density, cyclability, and voltage enhancement. Phys. Chem. Chem. Phys. 17, 2127–2136 (2015). 25484001 10.1039/c4cp04895d

[b35] Zheng J. M. . Lewis Acid-Base Interactions between Polysulfides and Metal Organic Framework in Lithium Sulfur Batteries. Nano Lett. 14, 2345–2352 (2014). 24702610 10.1021/nl404721h

[b36] Wagner C. D., Riggs W. M., Davis L. E., Moulder J. F. & Mullenberg G. E. Handbook of X-ra23y photoelectoron spectroscopy. Perkin-Elmer Corp., Physical Electronics Division, Eden Prairie, Minnesota, USA (1979).

[b37] Seh Z. W. . Facile synthesis of-Li_2_S-polypyrrole composite structures for high-performanceLi_2_S cathodes. Energy Environ. Sci. 7, 672–676 (2014).

[b38] Brown J. W. . Photophysical pore control in an azobenzene-containing metal-organic framework. Chem. Sci. 4, 2858–2864 (2013).

[b39] Lu G. . Imparting functionality to a metal-organic framework material by controlled nanoparticle encapsulation. Nat. Chem. 4, 310–316 (2012). 22437717 10.1038/nchem.1272

