# Supplementary material for: Encapsulation of redox polysulphides via chemical interaction with nitrogen atoms in the organic linkers of metal-organic framework nanocrystals
Source: Sci Rep. 2016 May 5;6:25555. doi: 10.1038/srep25555 (PMC4857742; doi:10.1038/srep25555)
Supplement: Supplementary Information [file srep25555-s1.doc]

Supporting Information for

**Encapsulation of redox polysulphides via chemical interaction with nitrogen atoms in the organic linkers of metal-organic framework nanocrystals**

Jung Hyo Park^1‡^, Kyung Min Choi^1,2‡^, Dong Ki Lee^1^, Byeong Cheul Moon^3^, Sang Rim Shin^1^, Min-Kyu Song^4*^ and Jeung Ku Kang^1,3*^

^1^Department of Materials Science and Engineering, Korea Advanced Institute of Science and Technology

291 Daehak-ro, Yuseong-gu, Daejeon 34141, Republic of Korea

^2^ Department of Chemical and Biological Engineering, Sookmyung Women’s University

Cheonpa-ro 47-gil 100, Yongsan-gu, Seoul 04310, Republic of Korea

^3^Graduate School of EEWS (Energy, Environment, Water, and Sustainability)

Korea Advanced Institute of Science and Technology

291 Daehak-ro, Yuseong-gu, Daejeon 34141, Republic of Korea

^4^School of Mechanical and Materials Engineering, Washington State University

Pullman, Washington 99164-2920, USA

^‡^Contributed equally to this work.

*Corresponding authors: minkyu.song@wsu.edu and jeung@kaist.ac.kr

**Table of Contents**

**Section S1.** Methods details

**Section S2.** Experimental characterizations

**Section S3.** Supplementary figures

**Section I.** Methods details

**S1. 1. Synthesis of nMOF-867.**

All of the chemicals have been purchased from Sigma Aldrich without further purifications. Zirconium chloride (≥ 99.9%) of 9.32 mg and acetic acid (≥ 99.7%) of 1.38 mL were dissolved in 5 mL of *N,N*-dimethylformamide (DMF, ≥ 99.8%). Also, 9.25 mg of 2.2′-bipyridine-5,5′-dicarboxylic acid (≥ 97%) and 35 µL of triethylamine (≥ 99.5%) were dissolved in 5 mL of DMF. Each solution was combined in the 20 mL glass vial. Next, the glass vial was sonicated in the water batch for 10 min. The combined solution became transparent from blurry solutions. The glass vial was then placed in the heating oven at 85 °C for 12 hrs. The white products were precipitated and collected with the centrifuge at 8000 rpm for 10 min. They were washed three times with DMF and methanol and immersed in methanol for three days. Finally, the products were activated in the vacuum oven 100 °C for 24 hrs. The fabricated nMOF-867 was kept in the Ar-filled glove box to protect the contact with the moisture in air.

**S1. 2. Synthesis of nUiO-67.**

All of the chemicals have been purchased from Sigma Aldrich without further purifications. Zirconium chloride (≥ 99.9%) of 18.64 mg and acetic acid (≥ 99.7%) of 1.38 mL were dissolved in 5 mL of *N,N*-dimethylformamide (DMF, ≥ 99.8%). Also, 19.36 mg of 4,4′-biphenyldicarboxylate (≥ 97%) and 30 µL of triethylamine (≥ 99.5%) were dissolved in 5 mL of DMF. Each solution was combined in 20 mL glass vial. Next, the glass vial was sonicated in the water batch for 10 min. The combined solution became transparent from blurry solutions. The glass vial was then placed in the heating oven at 85 °C for 6 hrs. The white products were precipitated and collected with the centrifuge at 8000 rpm for 10 min. They were washed three times with DMF and methanol and immersed in methanol for three days. Finally, the products were activated in the vacuum oven 100 °C for 24 hrs. The fabricated nUiO-67 was kept in the Ar-filled glove box to protect the contact with the moisture in air.

**I-3. Preparation of nMOFs/sulphur composite.**

The activated nMOF-867 or nUiO-67 was mixed with a pure sulphur (99.998%, Sigma Aldrich) in the mortar for 20 min. Next, we put the mixture into a tightly sealed vessel. It was put into the quartz tubular furnace using the Ar flow and heated up to 155 °C with the heat rate of 1°C/min for 12 hrs. Finally, the temperature was cooled down to room temperature and the sealed vessel was took out. These whole processes were conducted in the Ar-filled glove box.

**I-4. Preparation of nMOF-867/Li_2_S_4_ composites.**

For making the chemical interaction between nitrogen atoms and Li_2_S_4_ polysulphides, we artificially fabricated the Li_2_S_4_ polysulphides solution mixed with 506 mg of sulphur powder (99.998%, Sigma Aldrich) and 238.16 mg of Li_2_S (99.9%, Alfa Aesar) into 15 g of tetraethylene glycol dimethyl ether (≥ 99%, Sigma Aldrich). Next, the solution was stirred at 45 °C for 12 hrs and diluted to the 0.1M solution using fresh TEGDME. Next, the 0.1M Li_2_S_4_ polysulphides solution was mixed with the activated nMOF-867 in the 20 mL glass vial and stirred at room temperature for 12 hrs. Finally, the white nMOF-867 was turned into the brown products and it was washed three times with methanol. These whole processes were conducted in the Ar-filled glove box.

**Section S2.** Experimental characterizations

**S2. 1. PXRD:** The powder X-ray data were collected using a Rigaku SmartLab *θ*-2*θ* diffractometer in the reflectance Bragg-Brentano geometry employing a Johansson type Ge(111) monochromator-filtered Cu K_α1_ radiation at 1200 W (40 KV, 30 mA) power and equipped with a high speed 1D detector (D/teX Ultra). The samples were held in a non-reflective holder stage that was cut from silicon, parallel to (510) direction and scanned by the speed of 2 °/min in the continuous mode.

**S2. 2. SEM:** The extremely mild conditions of low acceleration voltages (1 keV) in the gentle beam mode were used to avoid the damage on the samples during the SEM (JSM-7600F, JEOL) observation. All of nMOF and nMOF/S samples were prepared by the direct deposition of nMOF/acetone dispersion (1 mg/mL) on the carbon substrate that was heated on a hot plate (70 ºC).

**S2. 3. STEM EDX:** For the STEM EDX observation (JEM-ARM200F produced by JEOL), the nMOFs/S was sonicated for 20 min and then dispersed with acetone on a copper TEM mesh grid. The STEM EDX images were obtained by Bruker Quantax 4000 to map the zirconium, carbon, nitrogen and sulphur elements.

**S2. 4. FT-IR:** For the high resolution FT-IR spectra, the 20 mg of a pristine nMOF-867 was mixed with the artificially fabricated 10 mL of Li_2_S_4_ polysulphides solutions in the vial glass for 12 hrs at room temperature. After a stirring process, the products were collected using the centrifuge (8000 rpm, 10 min). Then, it was washed three times with methanol and dried in the vacuum oven for 12 hrs at 60 °C. The dried brown powders were put into a customized vacuum chamber equipped with the ART reflector of FT-IR (FT-IR 6100, JASCO).

**S2. 5. Gas adsorption analysis:** The gas adsorption analysis was performed on a Quadrasorb automatic volumetric instrument. The liquid nitrogen bath (77 K), the ultra-high purity grade nitrogen, and the helium were used for the adsorption experiment. The samples were pretreated by the evacuating process at 100 °C for 24 hrs. Next, the sample was filled with the helium gas. Moreover, the apparent surface areas were determined by the BET method using the adsorption branches of the N_2_ isotherms.

**S2. 6. TGA measurements:** The TGA (TG 209 F1 Libra, NETZSCH) was measured to determine the thermal stability of nMOFs and the content of sulphur in the nMOF/S. The sample was put into the crucible and evacuated in the column. Next, the temperature was heated up to 700 °C (heat rate, 5 °C/min) in the nitrogen flow.

**S2. 7. XPS measurements:** The XPS spectra were obtained by using the K-alpha instrument (Thermo Scientific) equipped with the Al Ka micro-focused X-ray monochromator (1487 eV). The samples were prepared on the carbon tape, which was settled on aluminum metal plate, to reduce the electron charging on the surface of samples. The analysis chamber is precision- machined from a single billet of the nickel-iron alloy. This provides excellent magnetic shielding and the precision for alignment of the components.

**S2. 8. UV-visible spectroscopy:** The absorbance spectra were determined by a Cary-300 UV-visible spectrophotometer (VARIAN) with customized quartz cells sealed by using the septum. The 40 mg of nMOF-867 was put into the 0.1M Li_2_S_4_ TEGDME solution and stirred for 240 min. All of the preparations were carried out in the argon-filled glove box. And then, the solution was centrifuged at 8000 rpm for 5 min for sedimentation of the nMOF-867 and then the UV-visible spectroscopy was measured for supernatant solution every 30 min.

**S2. 9. *In-situ* spectroelectrochemistry measurements:** For measuring the *in-situ* spectroelectrochemistry, we have equipped the customized systems consisting of the light source (DH 2000, Ocean Optics) and the spectroscope (USB2000+, Ocean Optics). Simultaneously, the potentiosat (SP-300, Bio-Logics) was equipped with spectroscopic instruments for measuring the cyclic voltammetry (CV). Both of nMOF-867/S and nUiO-67/S were spin-coated on the Au coated quartz plate immersed into the lithium bis(trifluoromethanesulfonly)imide (1M) in 1:1 v/v 1,2-dimethoxyethane and 1,3-DOL containing LiNO_3_ (1 wt%), which were contained at quartz cuvette. The nMOFs/S composite was used as the working electrode while the Li metal foils were used as reference/counter electrodes. Also, the CV measurements were conducted under the sweep rate of 5 mV/s between 1.7 and 2.8V.

**Section S3.** Supplementary figures.

**
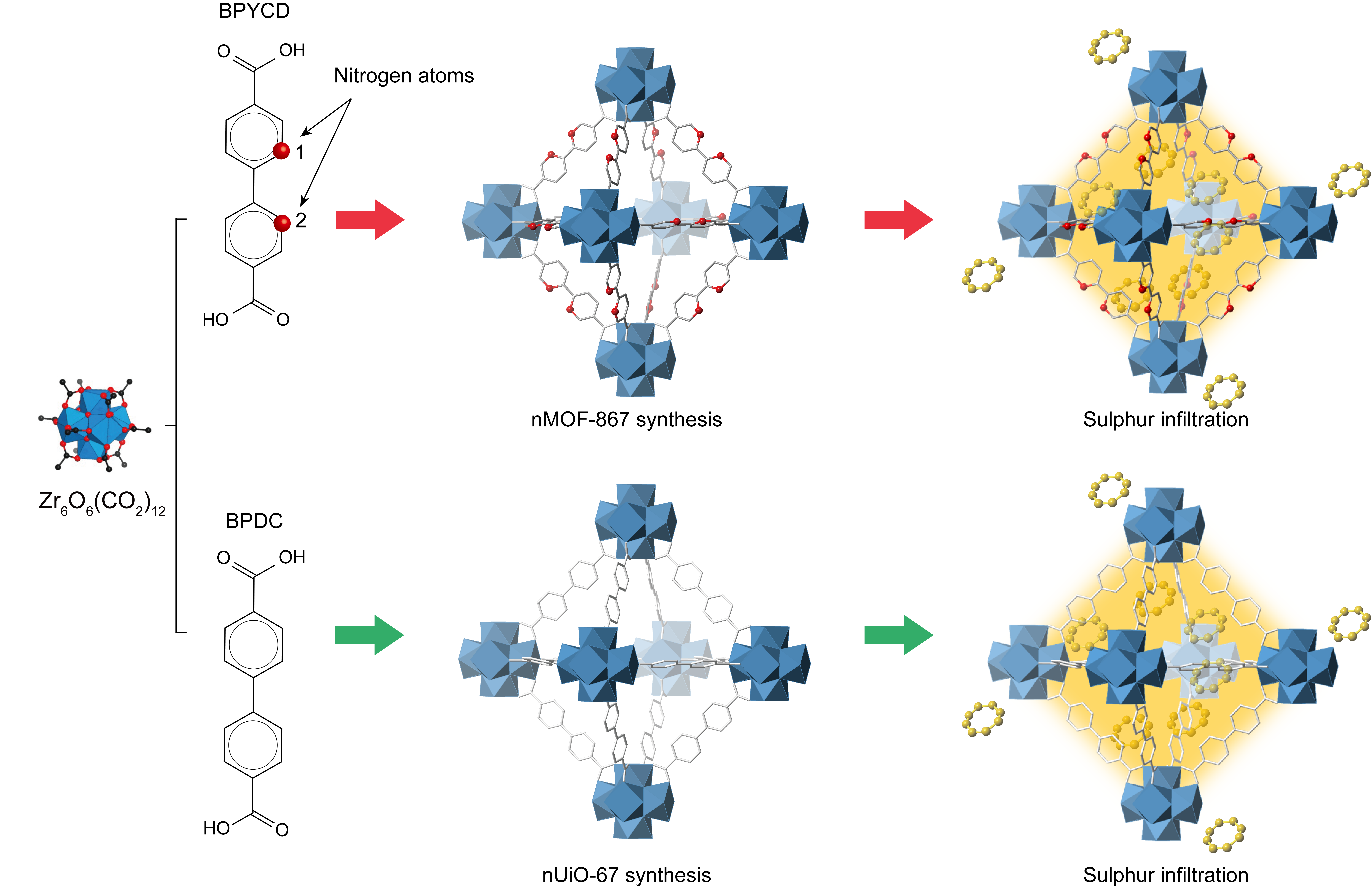
**

**Figure S1. Schematic fabrication process of nMOF-867 and nUiO-67. Next, the sulphur was infiltrated into the microcage of nMOF-867 and nUiO-67.** Both of nMOF-867 and nUiO-67 have the same crystal structure while their organic linkers were different. In the BPCD, the carbon atoms were embedded in the biphenyl group but the carbon atoms were replaced with sp^2^ nitrogen atoms embedded at position 1 and 2 as an atomic state in the BPYCD.


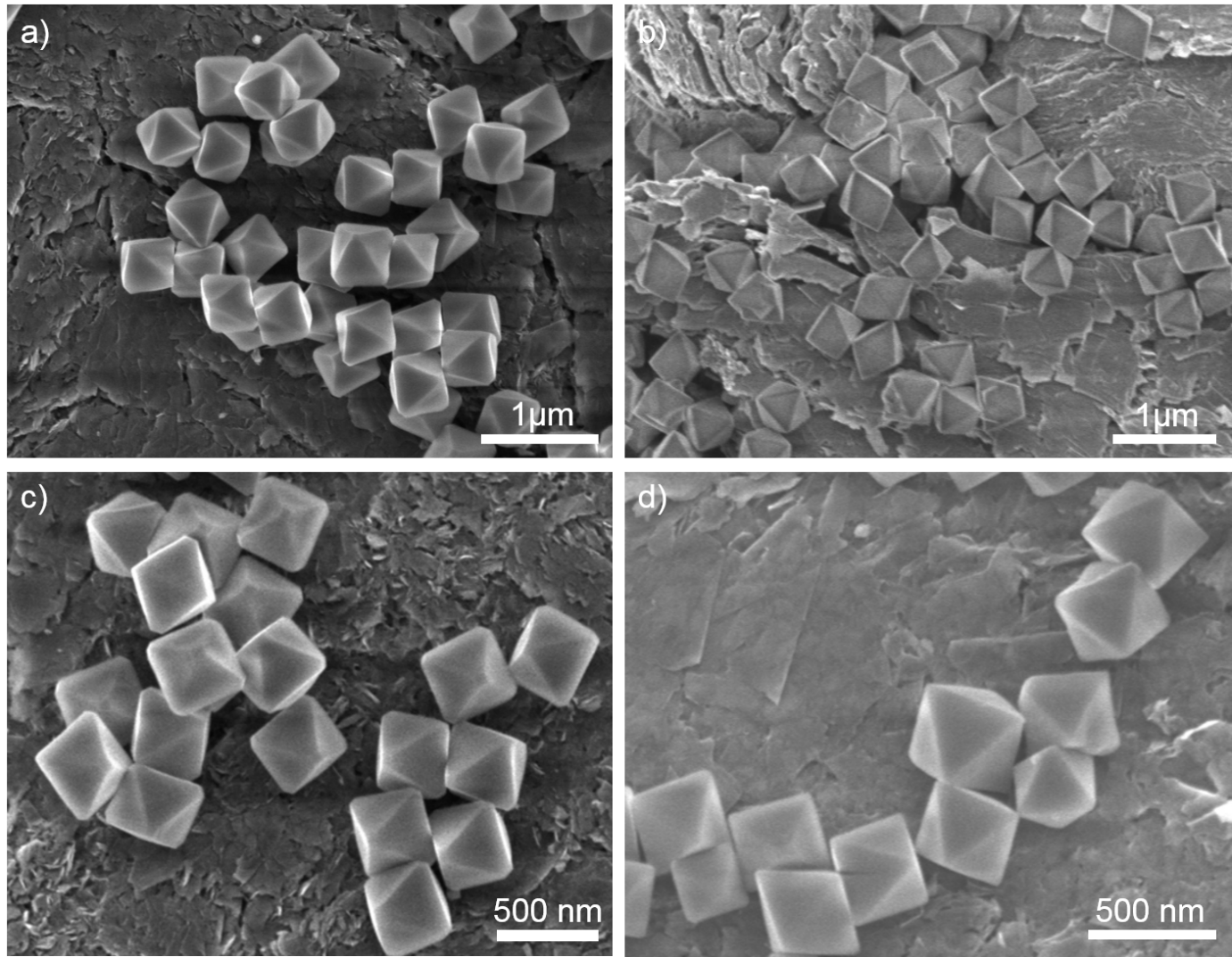


**Figure S2. SEM measurements of pristine nMOF-867 and nUiO-67.** The pristine structures for nMOF-867 (a and c) and nUiO-67 (b and d) have the similar morphologies of crystals and size about 500 nm.


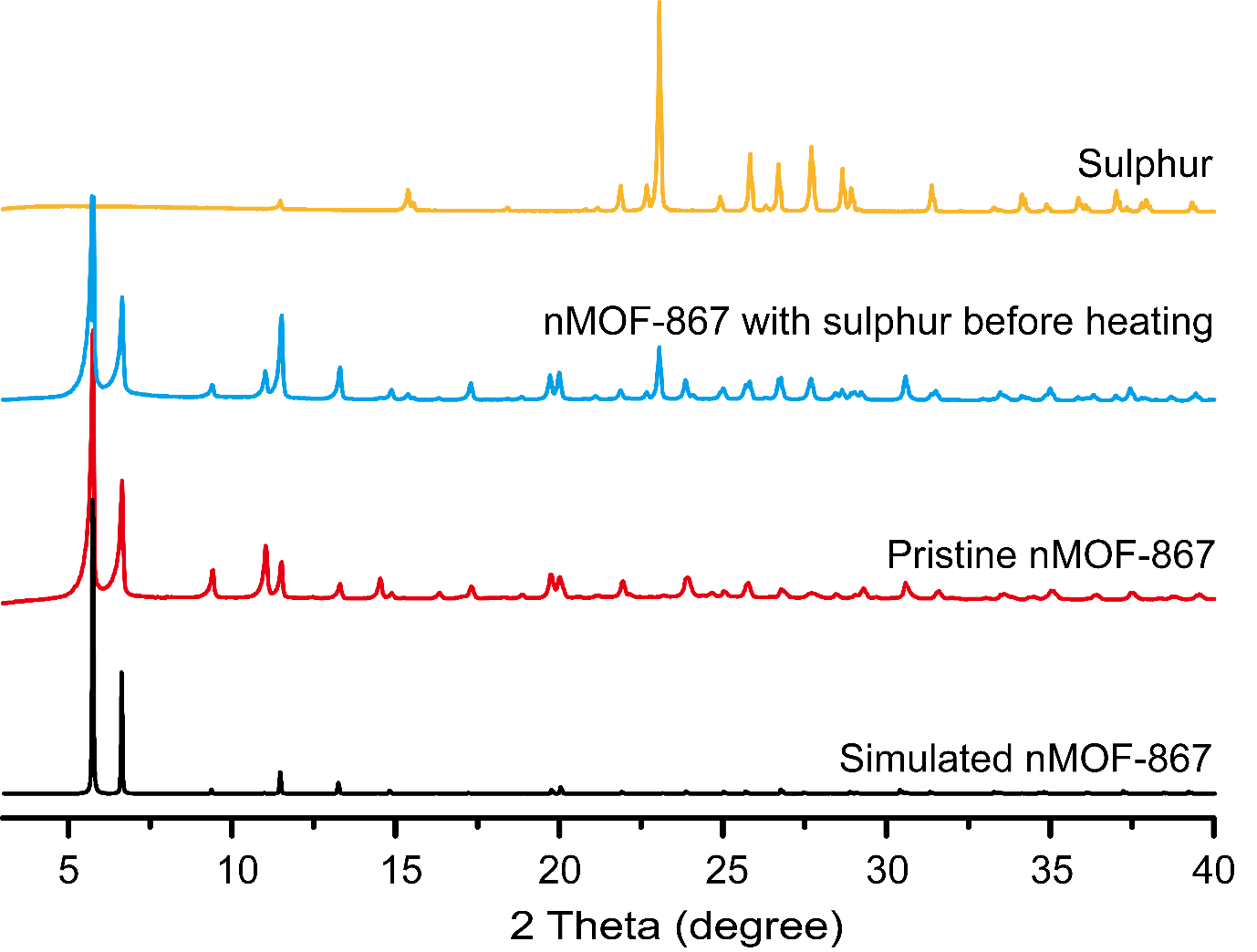


**Figure S3. PXRD patterns of sulphur particles, nMOF-867 with sulphur before heating, pristine nMOF-867 and simulated nMOF-867.** The pristine peaks for nMOF-867 are well-matched with the simulated patterns. The main peaks below 10° are related to the microcages of nMOF-867. Also, the PXRD patterns of the sulphur were well-matched with the orthorhombic phase structure (JCPDS card No. 08-0247). The sulphur shows the main peak at 23° and it is found that this peak for the sulphur is maintained in the case of nMOF-867 before heating.


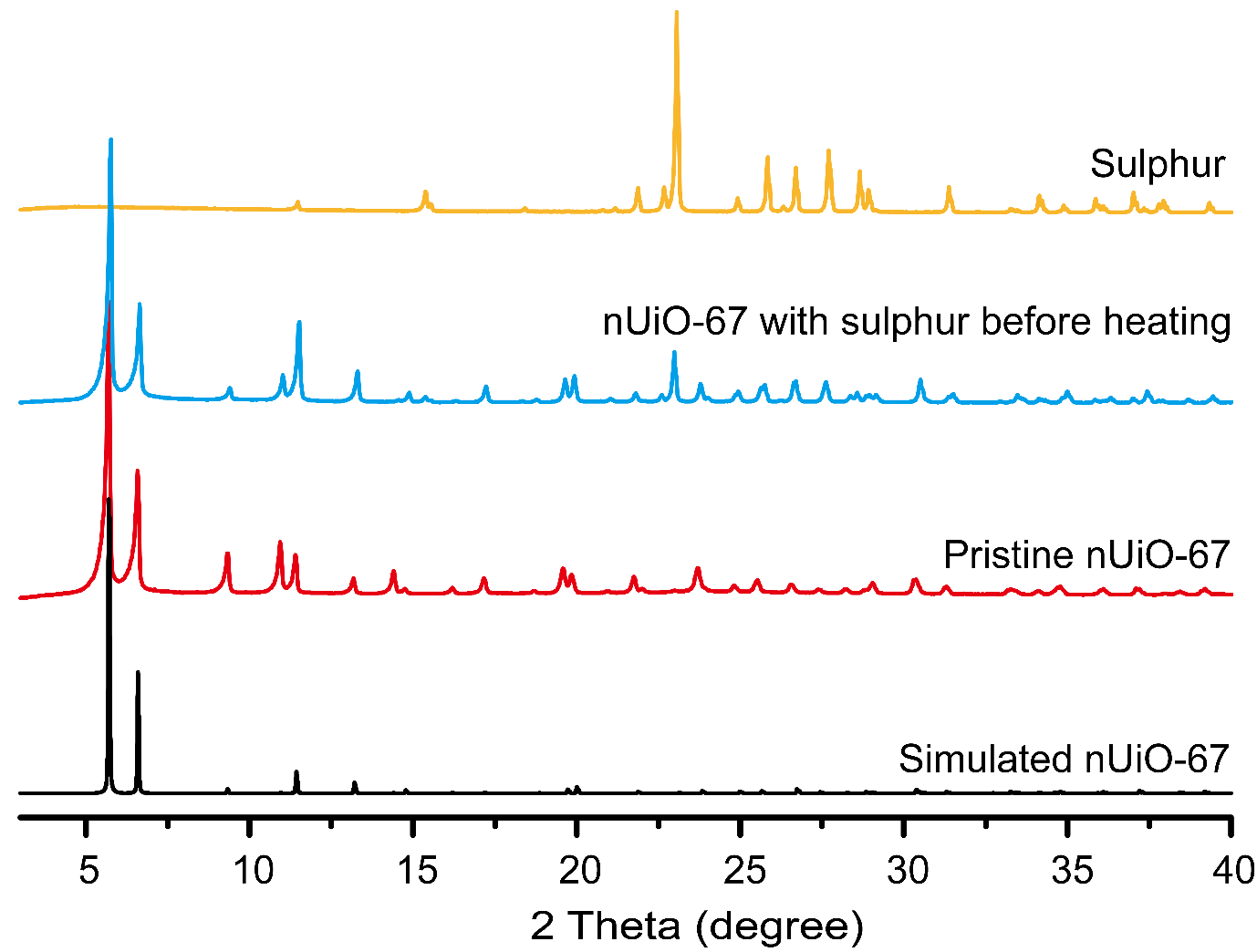


**Figure S4. PXRD patterns of sulphur particles, nUiO-67 with sulphur before heating, pristine nUiO-67 and simulated nUiO-67.** The pristine peaks for nUiO-67 are well-matched with the simulated patterns. These main peaks below 10° are related to the microcages of nUiO-67. The PXRD patterns of the sulphur are well-matched with the orthorhombic phase structure (JCPDS card No. 08-0247). The sulphur shows the main peak at 23° and it is found that this peak for the sulphur is maintained in the case of nUiO-67 before heating.

**
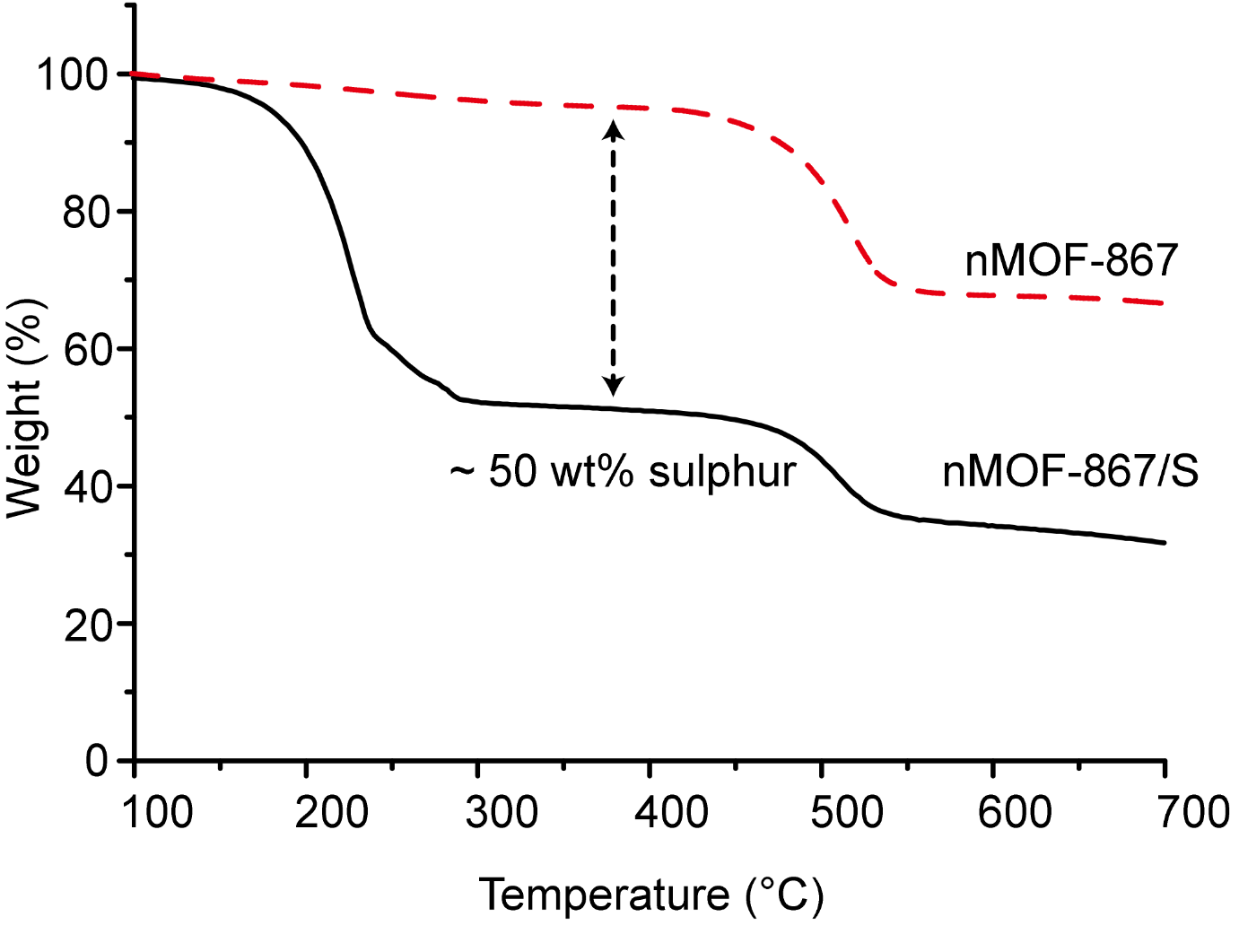
**

**Figure S5. Thermogravimetric measurement of pristine nMOF-867 and nMOF-867/S.** The TGA measurements for nMOF-867 and nMOF-867/S were conducted using the nitrogen flow rate of 5 °C/min. In case of nMOF-867, the drop of the weight percent was observed between 450 and 550 °C. Also, we have observed the drop of the weigh percent between 200 and 300 °C resulting from sublimation of the sulphur.

**
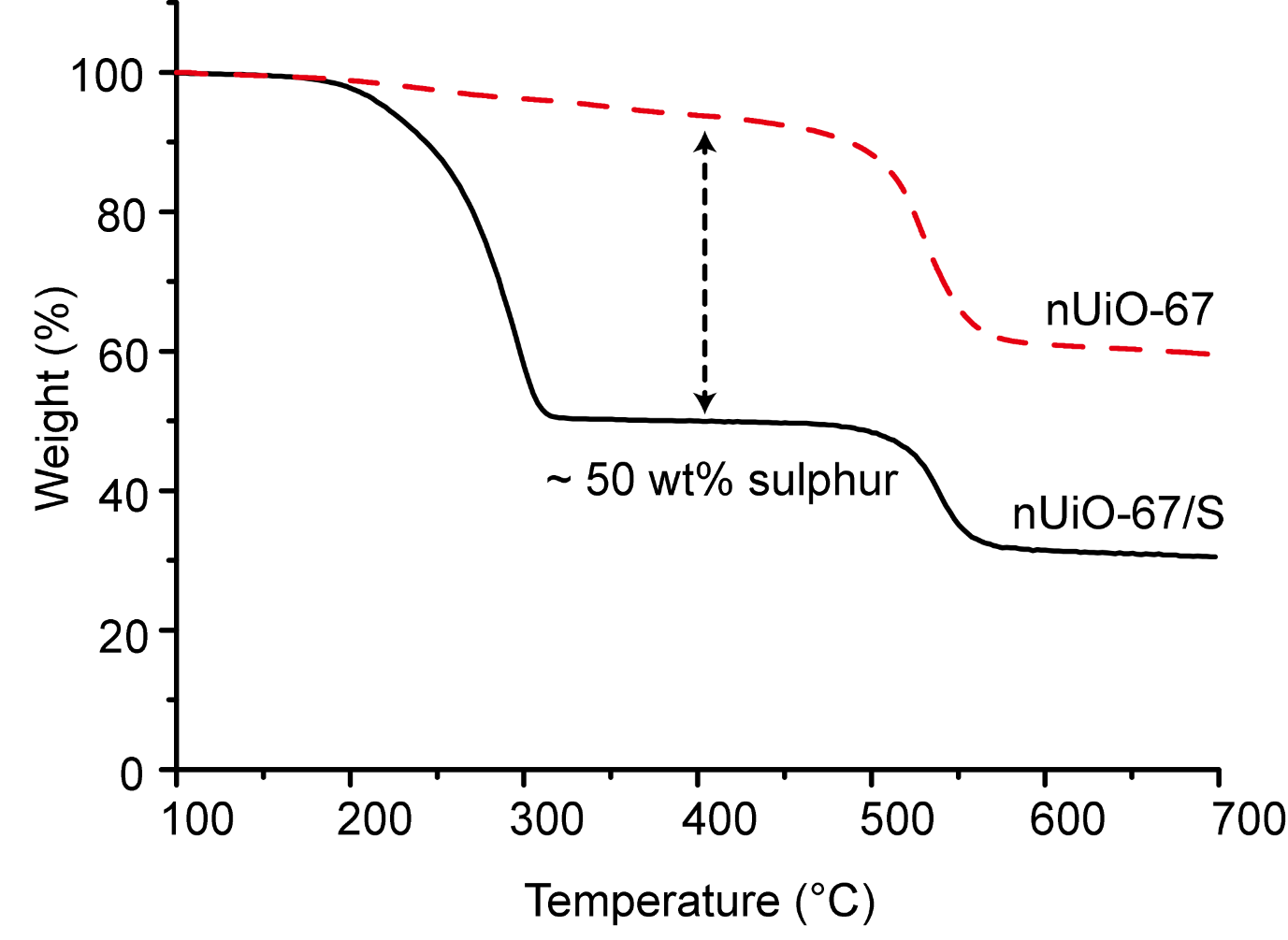
**

**Figure S6. Thermogravimetric measurement of pristine nUiO-67 and nUiO-67/S.** The TGA measurements for nUiO-67 and nUiO-67/S were conducted at the nitrogen flow rate of 5 °C/min. In case of nUiO-67, the drop of the weight percent was observed between 500 and 550 °C. Also, we have observed the drop of the weigh percent between 200 and 300 °C resulting from sublimation of the sulphur.


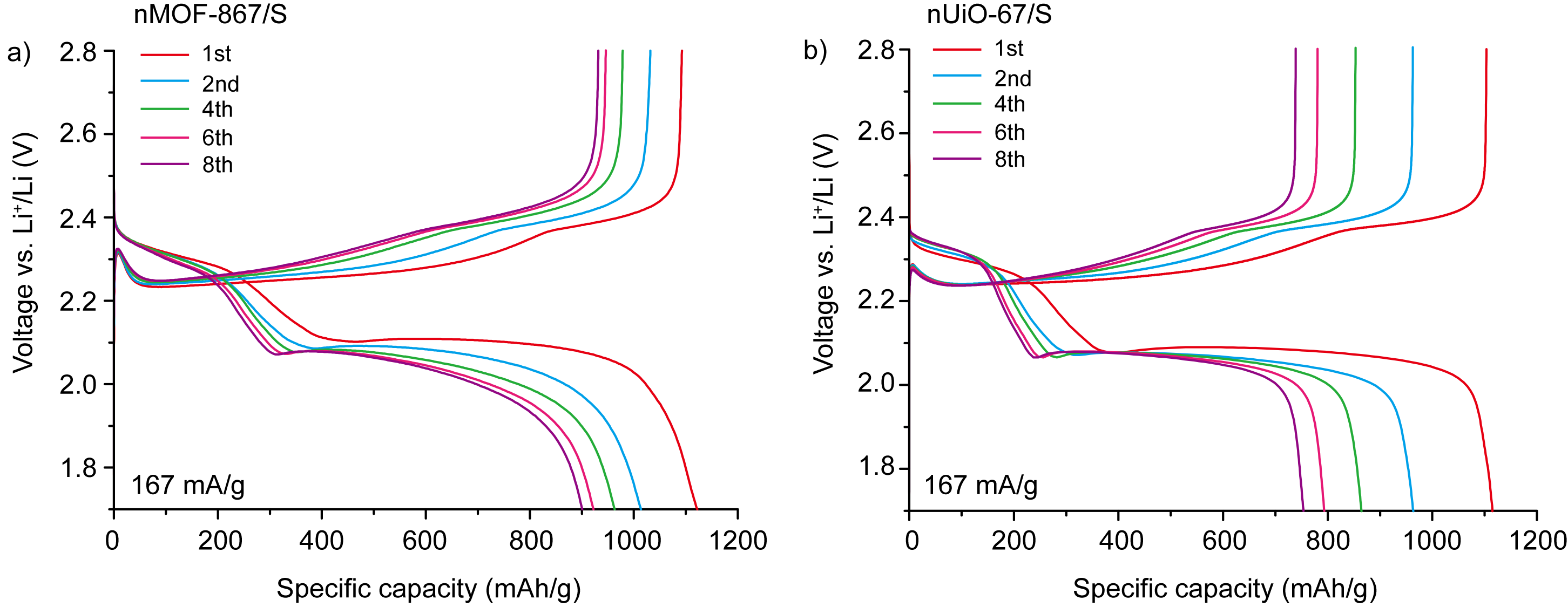


**Figure S7. The first few profiles of nMOF-867/S and nUiO-67/S at 167 mA/g.** a) 1^st^, 2^nd^, 4^th^, 6^th^ and 8^th^ discharge/charge profiles of nMOF-867/S. b) 1^st^, 2^nd^, 4^th^, 6^th^ and 8^th^ discharge/charge profiles of nUiO-67/S.


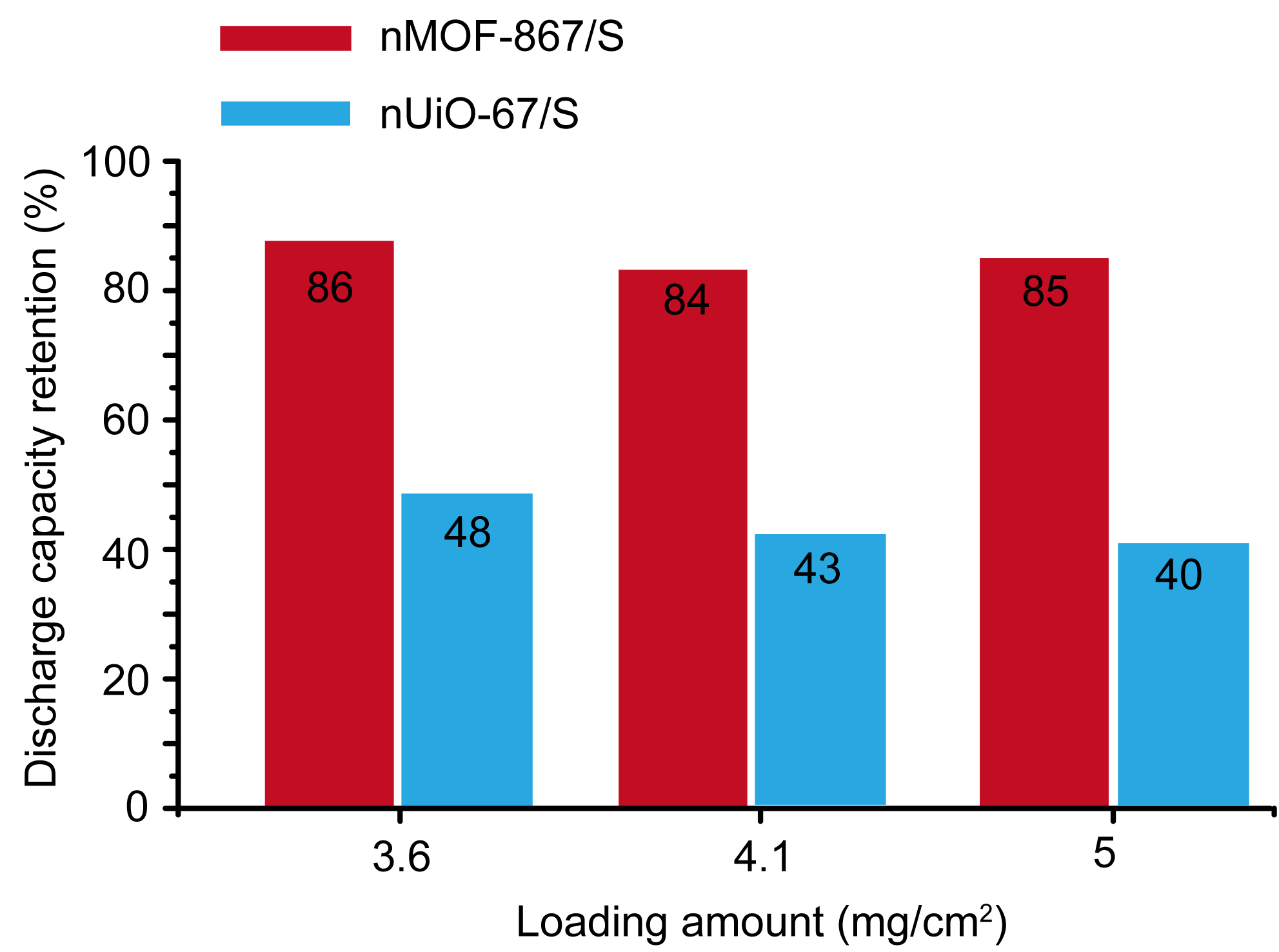


**Figure S8. Discharge capacity retention as different loading amounts of active materials.** We have varied the loading amounts of active materials and found that the discharge capacity retention of nMOF-867/S is maintained even on the increasing amounts of the active materials, although the discharge capacity retention of nUiO-67/S is gradually decreased.


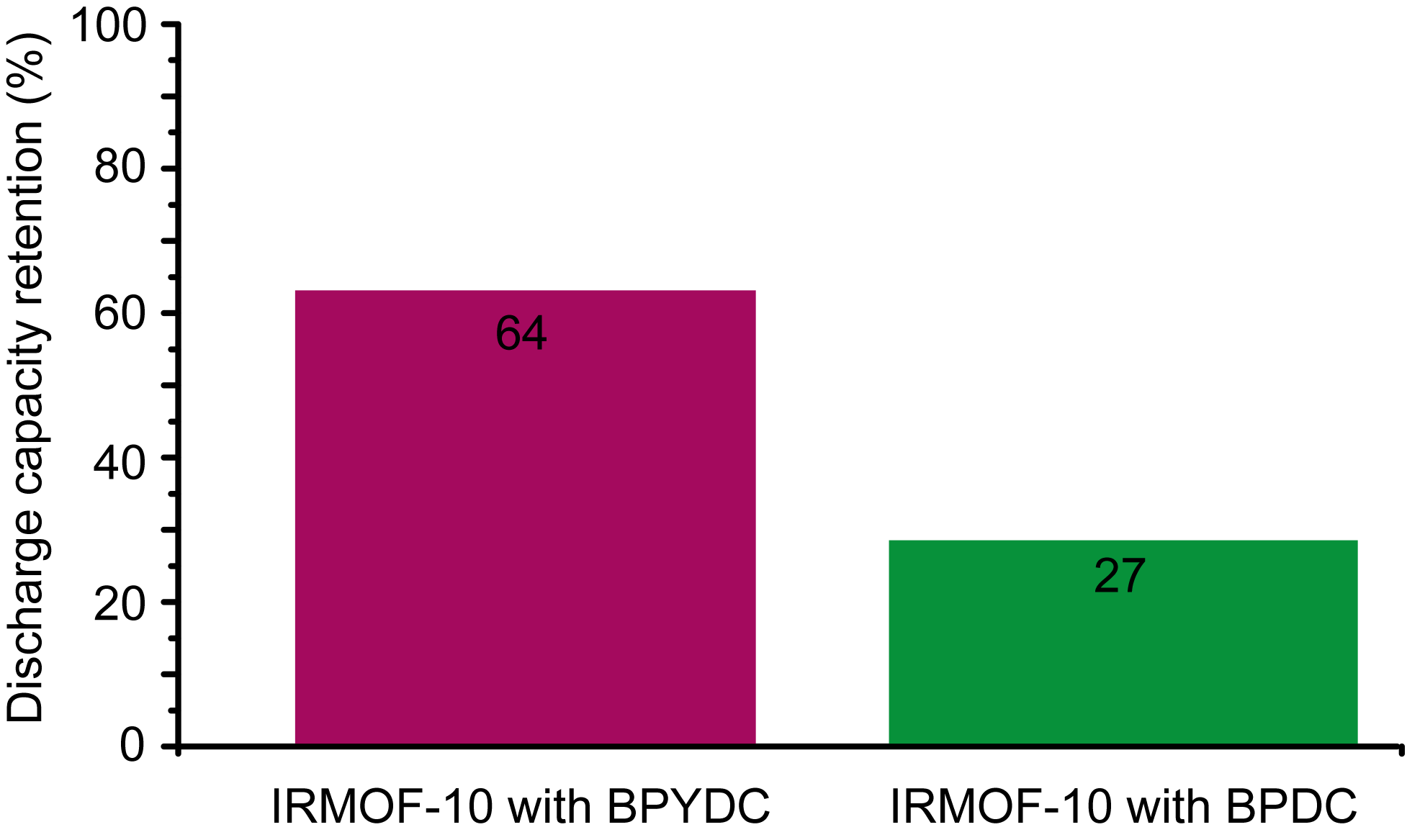


**Figure S9. The discharge capacity retention of IRMOF-10 with BPYDC and IRMOF-10 with BPDC.** a) IRMOF-10 with BPYDC has the 64% discharge capacity retention. b) IRMOF-10 with BPDC has the 27% discharge capacity retention.

**
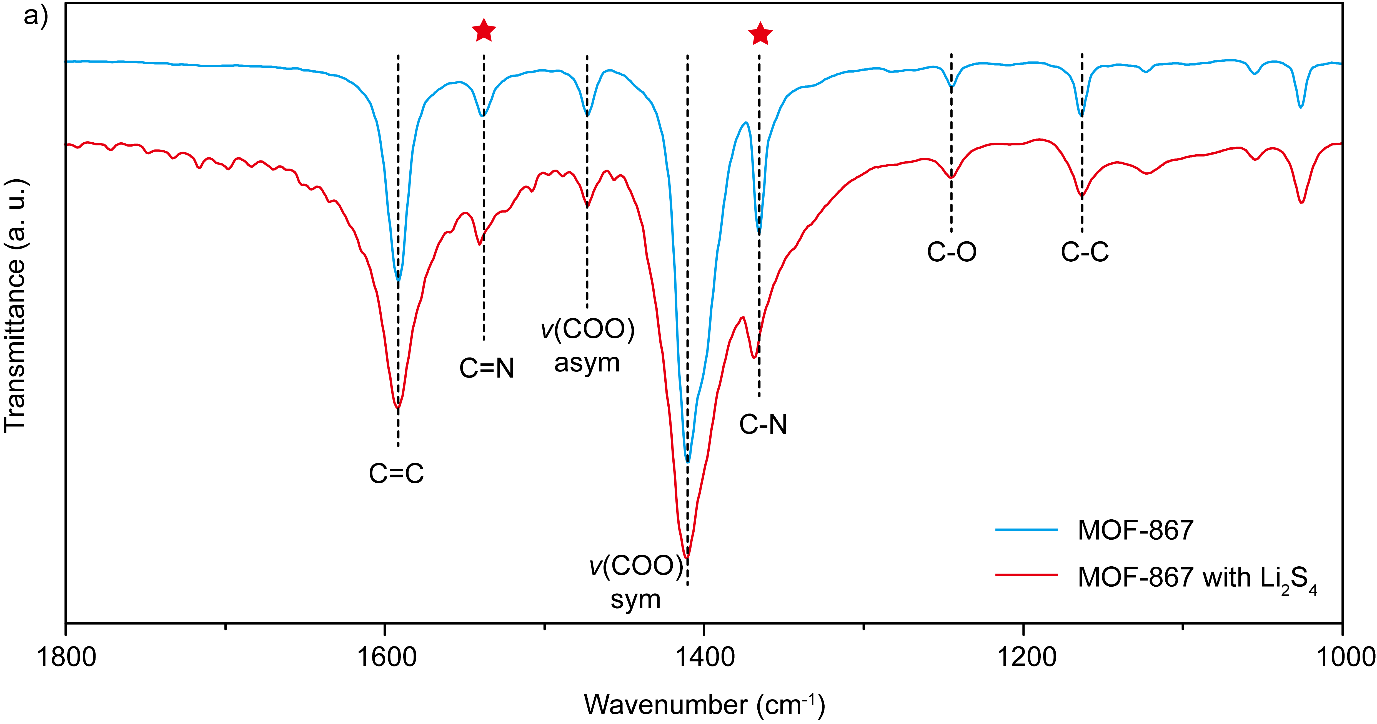
**

**Figure S10. FT-IR spectra of pristine nMOF-867 and nMOF-867 with Li_2_S_4_.** The FT-IR spectra were measured between 1800 and 1000 cm^-1^ and we found that the red star (★) peaks were shifted. This is attributed to the interaction of nMOFs with Li_2_S_4_ polysulphides. Meanwhile, the other peaks such as C=C, -COO-, C-O and C-C were found not to be shifted via their chemical interaction with Li_2_S_4_ polysulphides.


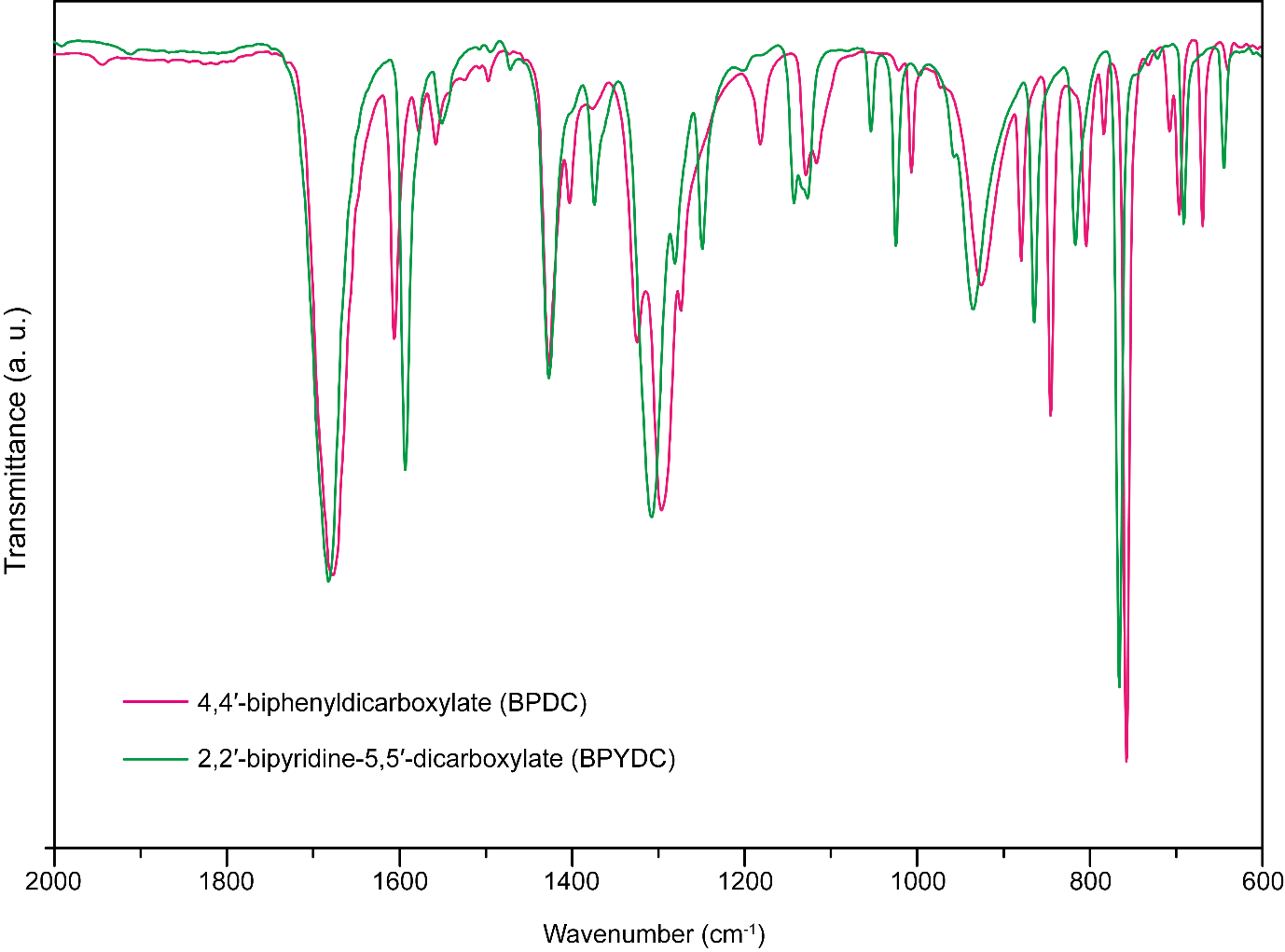


**Figure S11. FT-IR spectra for BPDC and BPYDC organic linkers.** The FT-IR spectra of organic linkers for nMOF-867 (BPYDC) and nUiO-67 (BPDC) show many sharp peaks. We find that the peaks related to C=C, C-C, C=N and C-N are maintained even after fabrication of nMOFs. However, it is determined that some other peaks have been disappeared.

**
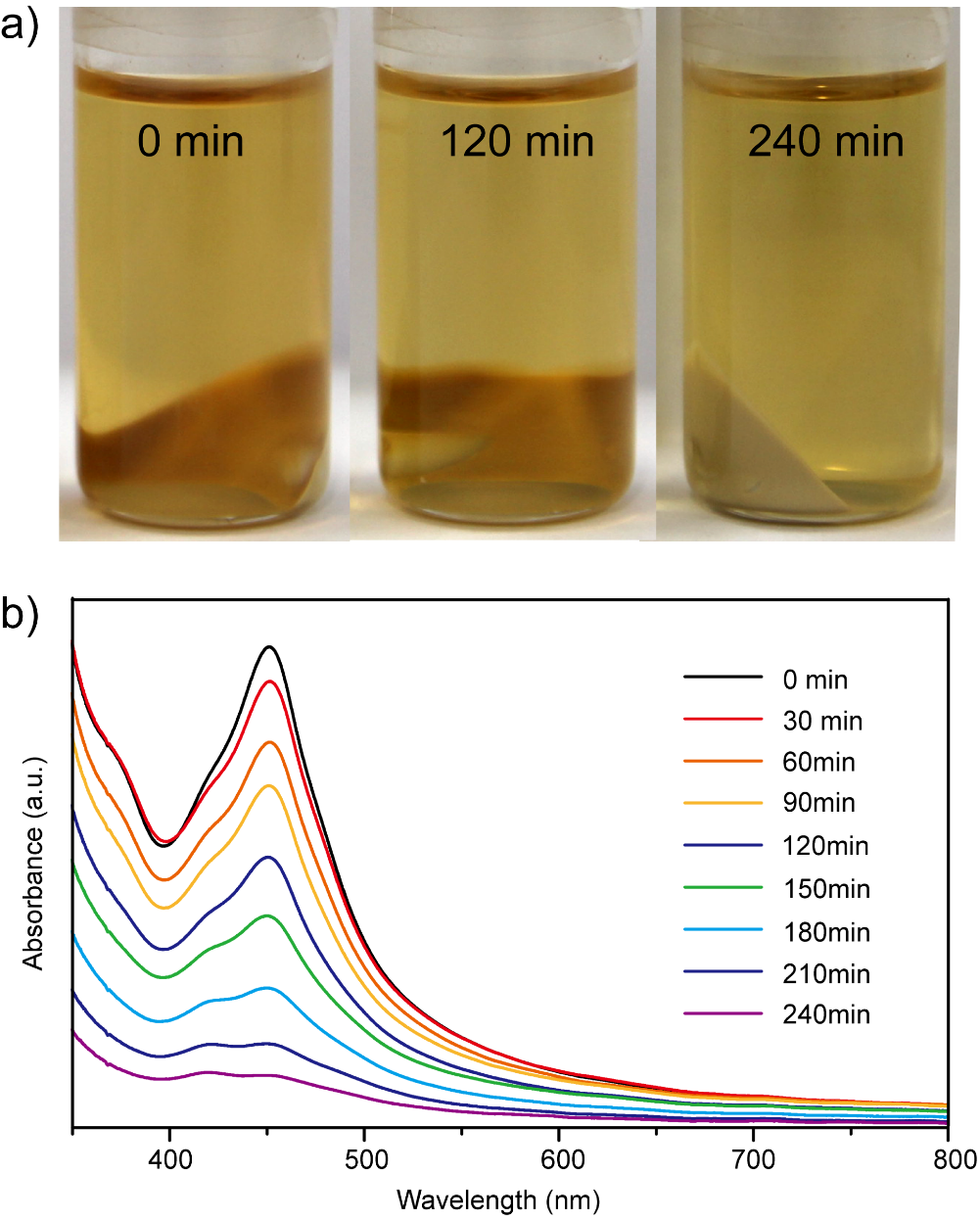
**

**Figure S12. Colour changes of nUiO-67/Li_2_S_4_ at 0 min, 120 min and 240 min and measuring the absorbance intensity of nUiO-67/Li_2_S_4_ every 30 min.** a) The photos show that the colour is still yellow at 240 min because the UiO-67 nanocrystals do not effectively attract the Li_2_S_4_ polysulphides. b) The absorbance intensity is slowly decreased as the photo observation.

**
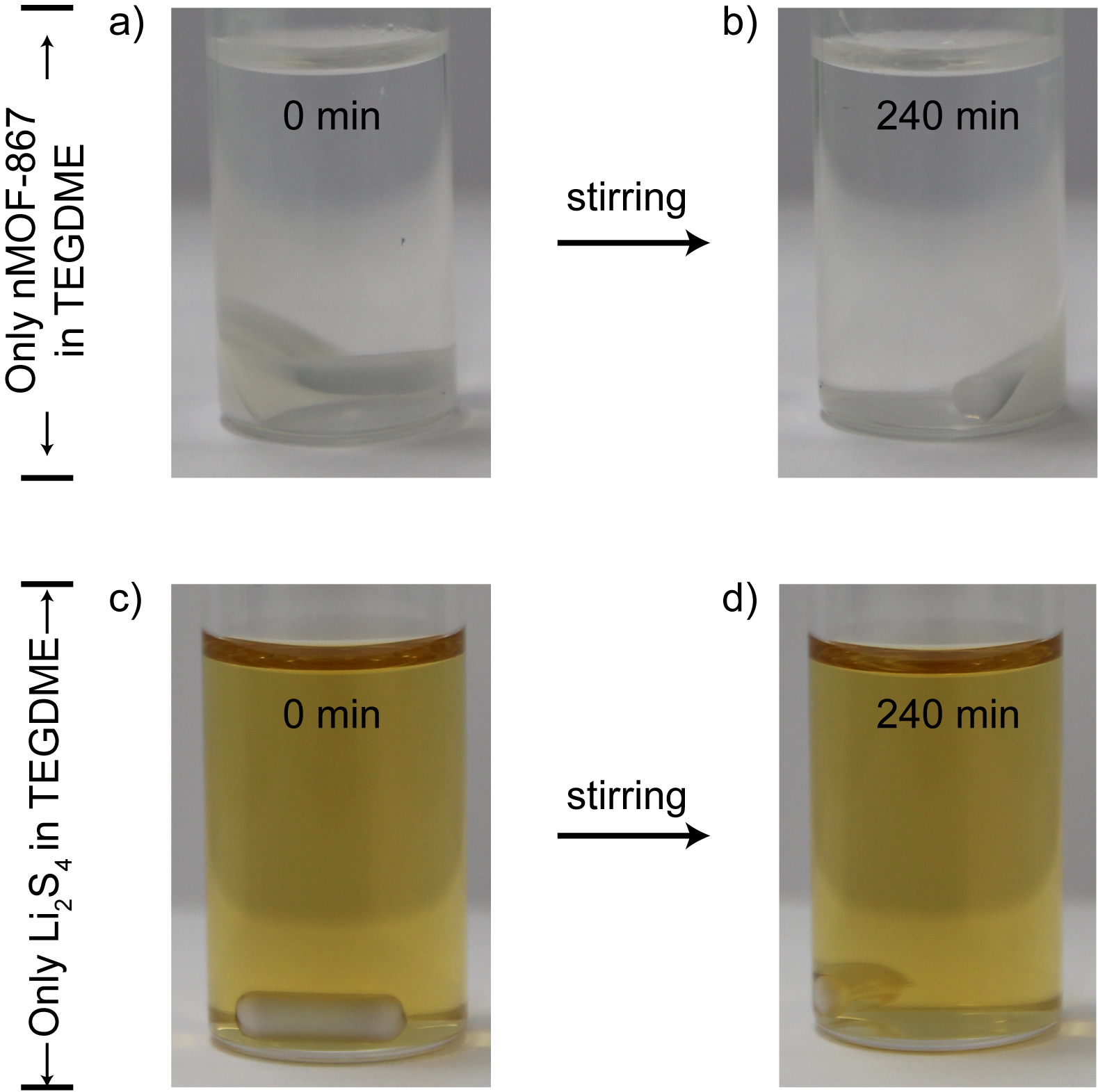
**

**Figure S13. The colour changes of nMOF-867 and Li_2_S_4_ in TEGDME solvent.** a) The solution of nMOF-867 with TEGDME is transparent at 0 min. b) The solution of nMOF-867 with TEGDME is also transparent and don’t have colour change at 240 min. c) The colour of only Li_2_S_4_ in TEGDME is yellow at 0 min d) The colour of only L_2_S_4_ in TEGDME is still yellow and don’t have colour change at 240 min.

**
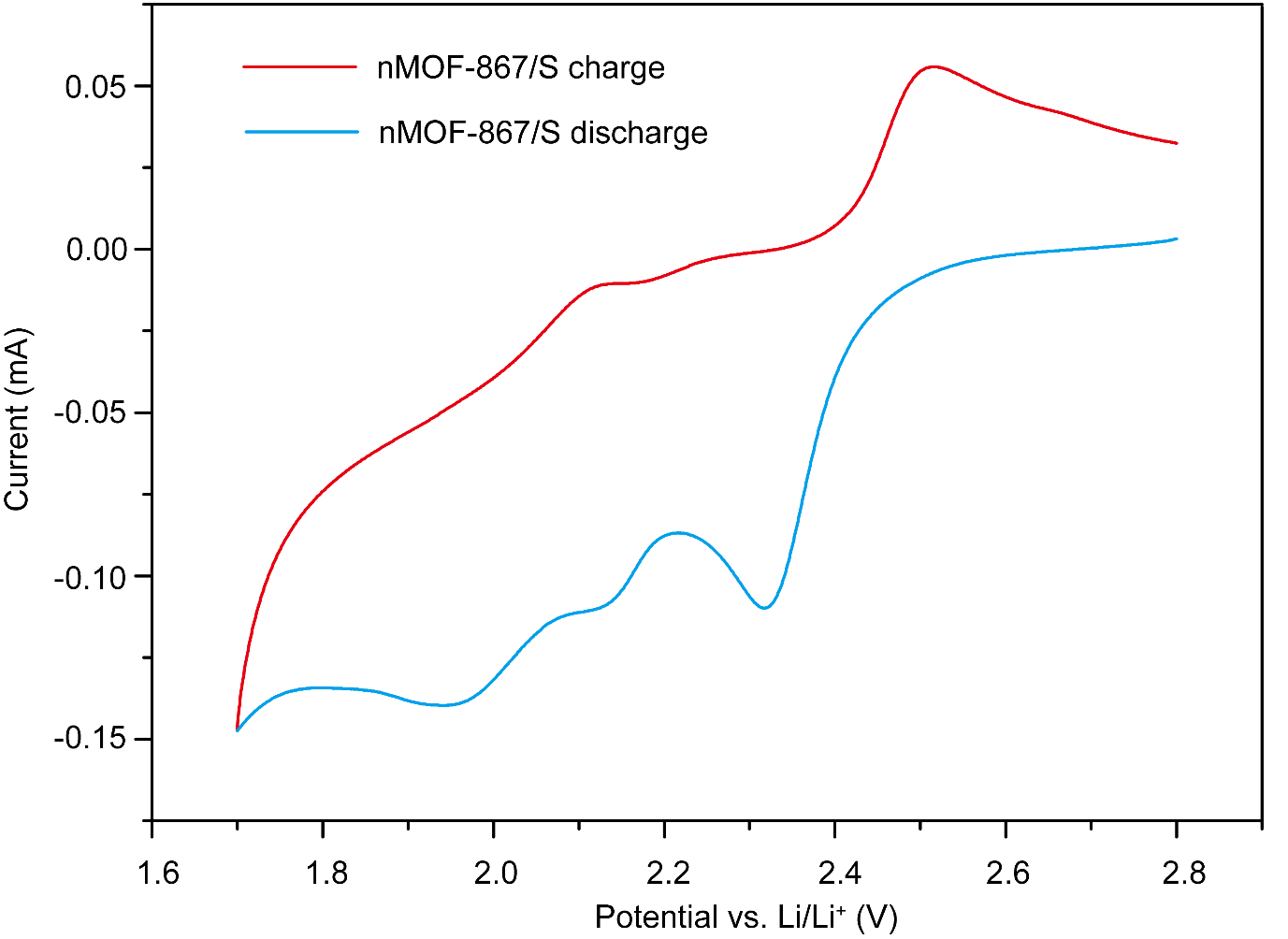
**

**Figure S14. Cyclic voltammetry (CV) measurements in the *in-situ* spectroelectrochemistry measurements of nMOF-867/S.**


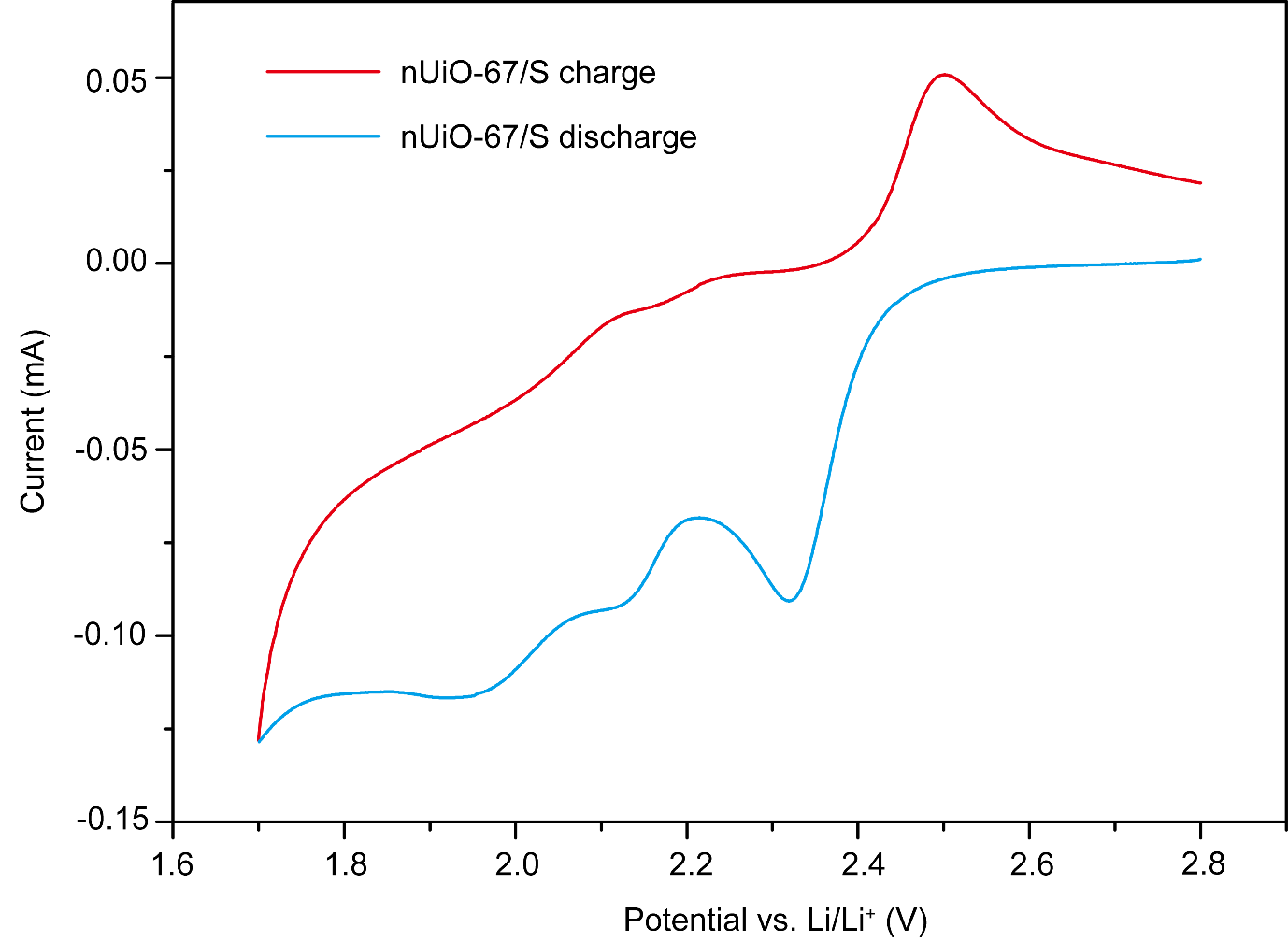


**Figure S15. Cyclic voltammetry (CV) measurements in the *in-situ* spectroelectrochemistry measurements of nUiO-67/S.**

In Figures S14and S15, we have observed the other side peaks in the charge/discharge reaction except for the peaks at 2.3 and 2.1V. This is because the *in-situ* spectroelectrochemistry measurements were conducted in the specific conditions. The working and counter electrode were immersed into the large quartz cuvette with a long distance between working and Li counter electrodes. (Figure 5a) To more clear this issue, we have also conducted the CV measurement as fabricating the coin cells using the same samples and we found that there were no other peaks in the charge/discharge reaction. (Figures S16 and S17)

**
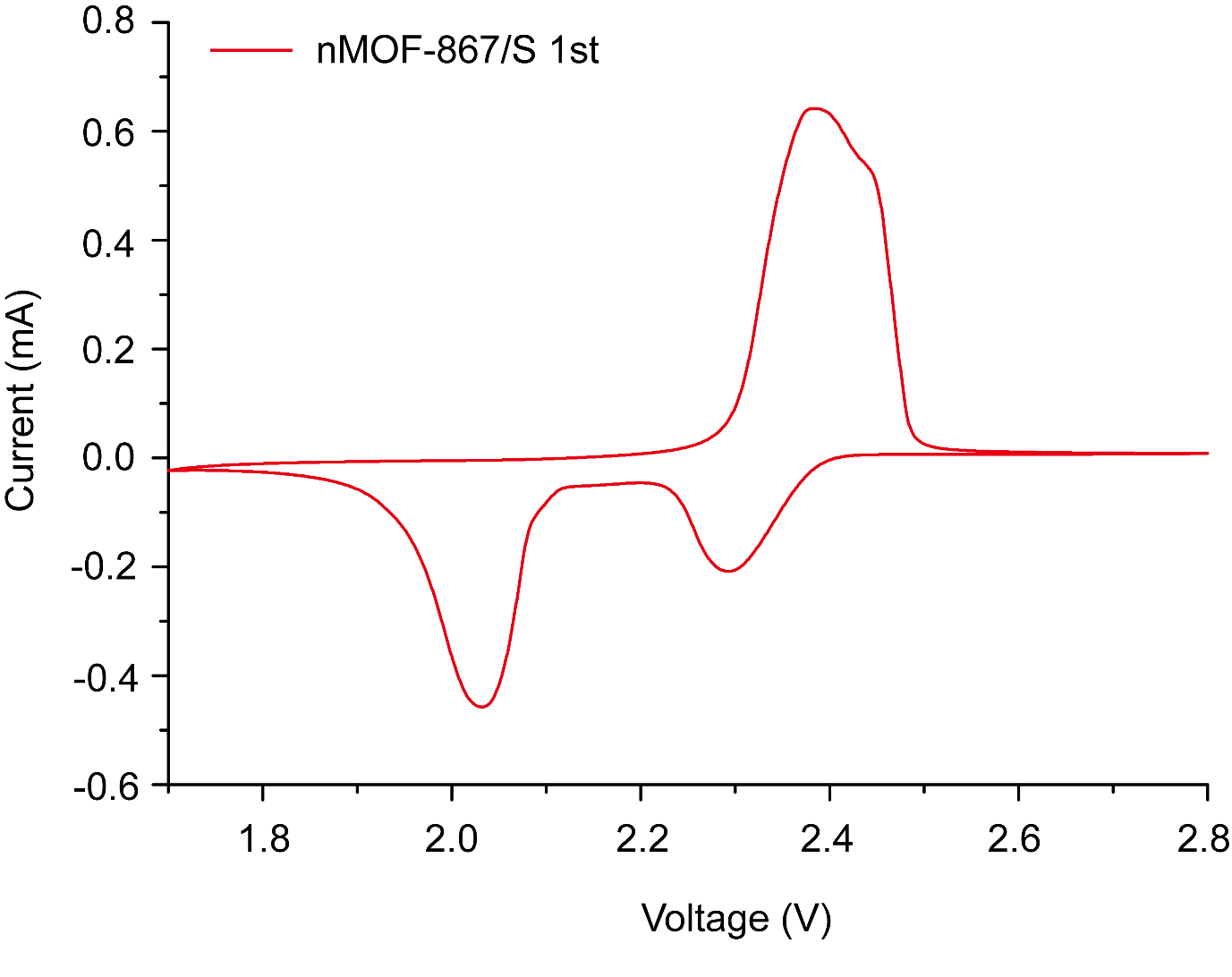
**

**Figure S16. Cyclic voltammetry (CV) of nMOF-867/S.** For the CV profile of nMOF-867/S at a scan rate of 0.1 mV/s in the potential range of 1.7 to 2.8 V vs Li/Li^+^, it has the two sharp peaks at 2.3 and 2.1 V during the discharge reaction, corresponding to the two plateaus in galvanostatic profiles.


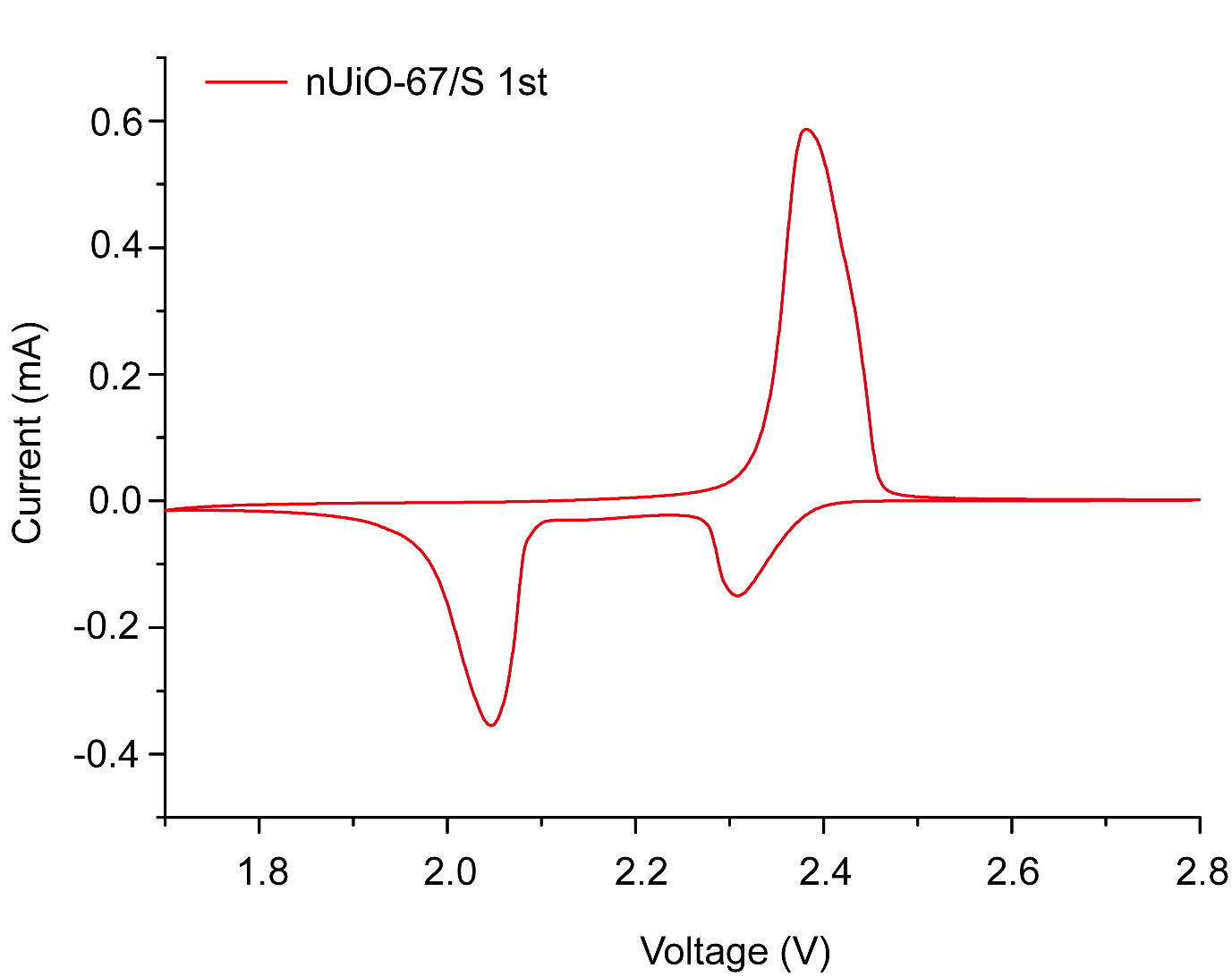


**Figure S17. Cyclic voltammetry (CV) of nUiO-67.** For the CV profile of nUiO-67/S at a scan rate of 0.1 mV/s in the potential range of 1.7 to 2.8 V vs Li/Li^+^, it has the two sharp peaks at 2.3 and 2.1 V during the discharge reaction, corresponding to the two plateaus in galvanostatic profiles.
